# Supplementary material for: Street images classification according to COVID-19 risk in Lima, Peru: a convolutional neural networks feasibility analysis
Source: BMJ Open. 2022 Sep 19;12(9):e063411. doi: 10.1136/bmjopen-2022-063411 (PMC9485648; doi:10.1136/bmjopen-2022-063411)
Supplement: Supplementary data [file bmjopen-2022-063411supp001.pdf]

Supplementary Materials

Street images classification according to COVID-19 risk in Lima, Peru: A convolutional neural networks feasibility analysis

Corresponding author:

Rodrigo M Carrillo-Larco, MD

Department of Epidemiology and Biostatistics

School of Public Health

Imperial College London

rcarrill@ic.ac.uk

Carrillo-Larco RM, *et al. BMJ Open* 2022; 12:e063411. doi: 10.1136/bmjopen-2022-063411

Contents

EXPANDED METHODS 3
Overview 3
System details 3
Images 3
Labels (outcome) 4
Class imbalance 4
Image data generator 4
Convolutional neural networks 5
Model Architecture 5
Model Training 6
Activation Heatmap by GradCam Visualization 7
References 7
Receiver Operating Characteristic (ROC) Curves I 9
Receiver Operating Characteristic (ROC) Curves II 10

EXPANDED METHODS

Overview

In a pre-specified protocol we decided to test five well-known convolutional neural network architectures. These five networks are those with the best accuracy among the models available in the Keras library.[1] From these five candidate models, we chose the one with the best performance for our task; this model was further tuned to improve its performance.

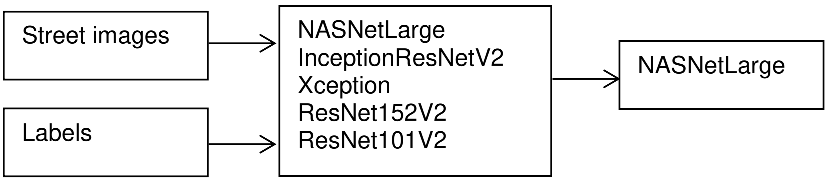

Analysis code (Jupyter notebooks) and the weights of the final model are found here: https://drive.google.com/file/d/1HXLsenn7yvxri7n2xE80WMtxQzj5fgBX/view?usp=sharing

System details

Analyses were conducted with a GPU NVIDIA Quadro P1000 on Python Jupyter (version 3.7.10). The notebooks are provided as supplementary materials.

Images

The list of bus stop in Lima, Peru, and their COVID-19 risk, are provided by local transport authority.[2] This information has been extracted and homogenized and is available online.[3] Key for our work, this information contains: i) location of each bus stop (latitude and longitude coordinates), which was used to extract street images; and ii) the COVID-19 risk level assigned to each bus stop, which was used as the outcome labels.

We downloaded the images from Google Street View through the application programming interface (API). We used the Python libraries *google_streetview.api* and *request*. For each bus stop (i.e., for each latitude and longitude coordinate), the API request specified the heading parameter = [0, 90, 180, 270]; in addition, we downloaded one image for which the heading parameter was set at default. Consequently, for each bus stop we had five images in total. The images were downloaded with size 640x640 pixels. In this feasibility study there were 1,788 bus stops, and because we had five images per bus stop, we used 8,940 (1,788 x 5) images in total.

Labels (outcome)

We used the bus stop classification released on 2021-05-24,[2, 3] by the local transport authority in Lima, Peru.[2] They classify the bus stops in four categories of COVID-19 risk: moderate, high, very high and extreme risk.[2] Details on how they made this classification are not available. Nevertheless, because this is official information released by a public authority to inform the general population, we trust their classification is based on the best available evidence. In this feasibility work we only used bus stops labelled as *moderate* (n=1,173) and *extreme* (n=615) COVID-19 risk. There were 1,788 (1,173 + 615) bus stops in total. The list of labels was appended five times, so that we would have as many images as labels (NB: we had five images per bus stop as described above).

Class imbalance

There were two outcomes of interest: moderate and extreme risk. However, there were more observations in the moderate category than in the extreme category. That is, there was class imbalance. After splitting the data into the training, test and validation sets, we corrected for class imbalance. We randomly increased the number of observations in the extreme category by 90% in the training dataset (not in validation and test datasets). The original (before correction for class imbalance) training set had 3,519 observations in the moderate category and 1,845 in the extreme category (3,519 + 1,845 = 5,346). After correcting for class imbalance as described before, the training dataset had 3,519 observations in the moderate category (this number did not change) and 3,505 (~1.9 x 1,845) observations in the extreme category (3,519 + 3,505 = 7,024 total sample in the training dataset).

Image data generator

We constructed a dataframe with two columns: the path to each image (i.e., to the exact location were the images were saved) and the corresponding label for each image. This dataframe was passed to the *ImageDataGenerator* function of the *keras.preprocessing.function* library. At this point, we also re-scaled the images between 0 and 1 by dividing by 255. Then, we created three image iterators: one for the training, validation and test datasets (*train_datagen.flow_from_dataframe* function). To the *train_datagen.flow_from_dataframe* function we passed the datarame with the location of the images and their labels, specified this was a categorical classification problem, a batch size of 32, and a

specific image size for each candidate model (see table below); in addition, the image iterator for the training dataset had the shuffle parameter as *True*.

Convolutional neural networks

We decided to train five candidate convolutional neural networks with the following specifications.

	NASNetLarge	InceptionResNetV2	Xception	ResNet152V2	ResNet101V2
**Image size**	331 x 331	299 x 299		224 x 224	
**Pre-trained weights**	ImageNet				
**Top layer included?**	No				
**Trainable parameters**	None				
**Additional layers**	Dense layer with 2 neurons (for the two outcome labels), with *softmax* activation				
**Number of epochs for training**	25				
**Optimizer**	SGD(learning_rate = 0.1, momentum = 0.9, decay = 0.1/number of epochs, nesterov = True)				
**Loss function**	Binary crossentropy				

SDG: stochastic descent gradient.

In addition, we monitored the validation loss: when the validation loss would not improve in one epoch, then the learning rate was multiplied by 0.1. We also specified an early stop: when the validation loss would not improve for ten epochs, the training would stop. To choose between the five candidate models we did not implement any data augmentation methods.

We chose the model with the best performance (Table 1 in the main text), which was further tuned (Table 2 in main text).

Model Architecture

The chosen model (NASNetLarge) presented in this article is a state-of-the-art convolutional neural network (CNN) pre-trained with the ImageNet dataset of images. The NASNetLarge neural network is widely used in computer vision. It is composed of several layers of convolutional cells that extract the most important features from each image, learning which features are the most characteristic from each image category. Most pre-trained state-of-the-art CNNs, such as the AlexNet or the ResNet50, base their innovations in the use of complex activation functions, efficient designs and special layers, such as the Batch Normalization,[4] which not only improve accuracy performance, but also reduce the time needed to train a model. The ResNet50 neural network, for example, introduces the concept of residual neural networks, layers that skip their connections to other layers by adding connection shortcuts, thus reducing backpropagation training time and better generalizing models.

Carrillo-Larco RM, *et al. BMJ Open* 2022; 12:e063411. doi: 10.1136/bmjopen-2022-063411

However, most of these CNNs had complex design that was built by using trial-and-error methods, oblivious to modern state-of-the-art optimization methods such as the use of evolutionary and nature-inspired algorithms or Reinforcement Learning (RL). This is where Neural Search Architecture (NAS) Networks come in play.[5] Dubbed as a breakthrough in machine learning automation, NAS networks use *AI for AI.* The network's whole architecture is designed by optimization algorithms, such as Gradient-based search and RL. The idea behind this is that, setting the right restrictions to avoid repetitive inclusion of layers, the network cannot only be trained by its parameters, but by its own architecture, adding and changing layers, activations functions and connections.

Our research uses the NASNetLarge model available by Keras,[1] pre-trained on the ImageNet dataset on 1000 categories. Because we only have two categories to train on (moderate and extreme), we started by replacing the network's fully connected classification layer by a 2-neurons layer, with a softmax activation function.[1] The rest of the original NASNetLarge network was kept unmodified to preserve its proven architecture.

Model Training

To train our model we used transfer learning. Based on the pre-trained NASNet model, we retained its parameters and froze them, keeping just trainable the last fully connected layer, initializing its parameters randomly by He uniform initialization.[6] This single-stage training took advantage of the pre-trained parameters speeding up training by just focusing on the last decision layer. The model was trained for 30 epochs. Each epoch is understood as a complete training cycle through the whole train dataset. Data is then fed by batches; once all batches are loaded, an epoch is finished. By using a batch size of 32, training and testing sets are fed to the neural network. The loss function, as we are dealing with a binary classification model, is binary cross entropy.

As optimizer we used the Stochastic Gradient Descent (SGD).[7] One of the key advantages of this optimizer is due to its stochasticity in selecting each batch for the backpropagation step. Although the model takes longer to converge, unlike other optimizers, the SGD has been proven to converge better local optima, searching for global optima with much more easiness. This factor helps also to reduce overfitting.

Carrillo-Larco RM, *et al. BMJ Open* 2022; 12:e063411. doi: 10.1136/bmjopen-2022-063411

Activation Heatmap by GradCam Visualization

Model interpretability is a feature that has gained importance since the appearance of methods such as GradCam.[8] This technique uses the values from the gradients in the model's final feature layer to produce visual explanations, highlighting regions of importance taken by the model to infer a given input. In other words, this technique informs which areas of the input figure were more relevant to make the final classification.

Regions that represent higher gradient values, accounting for most of the last layer activations and network's decision, are represented by being coloured closer to the red portion of the spectrum. By comparison, regions with the lowest activations, not adding much information to the network's final decision, appear as areas closer to the blue portion of the spectrum. These gradient values are taken from the network's last convolutional layer, right before the last pooling layer.

References

1. Keras applications. URL: https://keras.io/api/applications/.

2. Autoridad de Transporte Urbano para Lima y Callao (ATU). Paraderos con Riesgo de COVID - 19. URL: https://sistemas.atu.gob.pe/paraderosCOVID.

3. URL: https://github.com/jmcastagnetto/lima-atu-covid19-paraderos.

4. Ioffe S, Szegedy C. Batch Normalization: Accelerating Deep Network Training by Reducing Internal Covariate Shift. *arXiv e-prints* 2015: arXiv:1502.03167.

5. Kyriakides G, Margaritis K. An Introduction to Neural Architecture Search for Convolutional Networks. *arXiv e-prints* 2020: arXiv:2005.11074.

6. He K, Zhang X, Ren S, Sun J. Delving Deep into Rectifiers: Surpassing Human-Level Performance on ImageNet Classification. *arXiv e-prints* 2015: arXiv:1502.01852.

7. Loshchilov I, Hutter F. SGDR: Stochastic Gradient Descent with Warm Restarts. *arXiv e-prints* 2016: arXiv:1608.03983.

8. Selvaraju RR, Cogswell M, Das A, Vedantam R, Parikh D, Batra D. Grad-CAM: Visual Explanations from Deep Networks via Gradient-Based Localization. *International Journal of Computer Vision* 2020; **128**(2): 336-59.

Receiver Operating Characteristic (ROC) Curves I

InceptionResNetV2

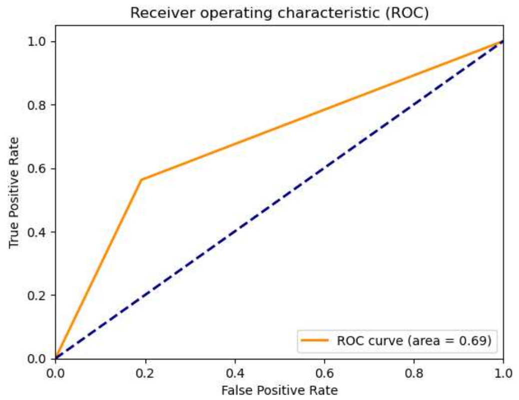

Xception

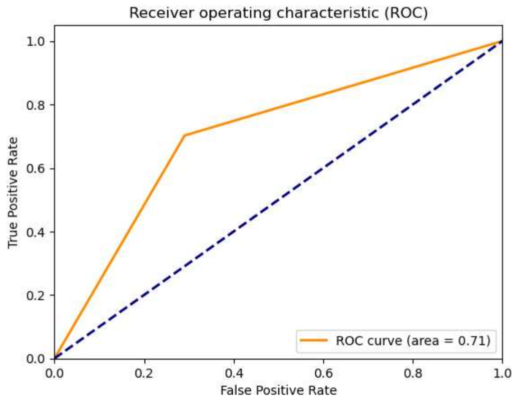

ResNet152V2

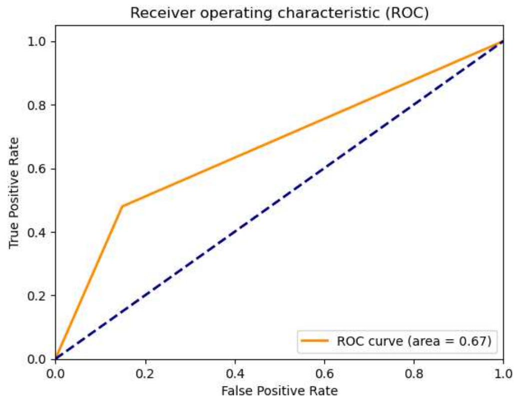

ResNet201V2

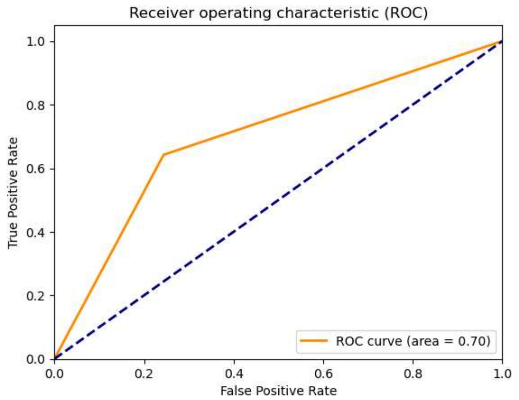

NASNetLarge

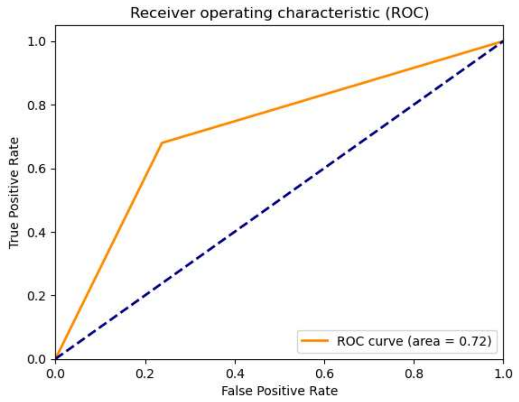

Receiver Operating Characteristic (ROC) Curves II

NASNetLarge [horizontal_flip = True // epochs = 25]

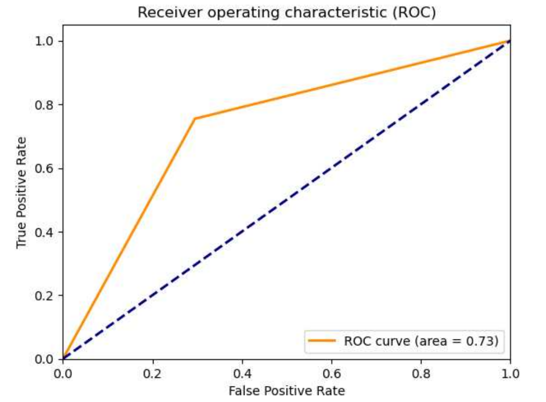

NASNetLarge [horizontal_flip = True // zoom_range = 0.30 // epochs = 25]

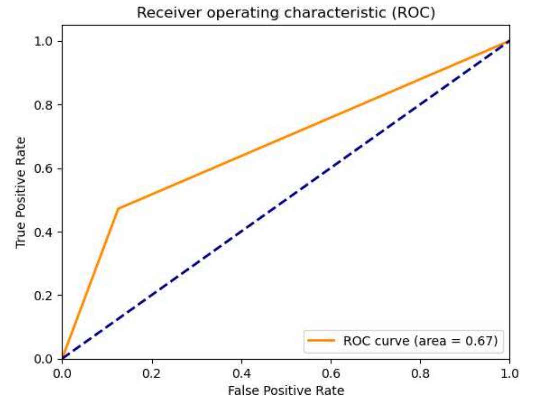

NASNetLarge [decay = 0.1/10 // epochs = 25]

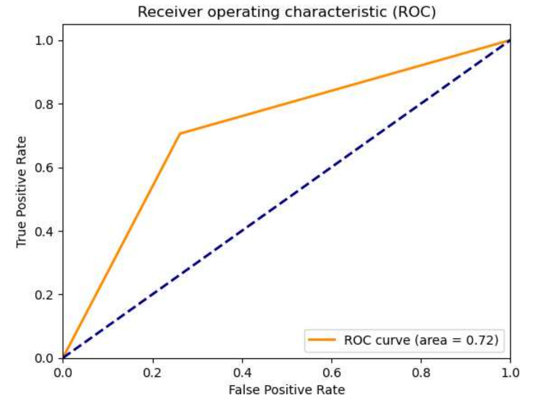

NASNetLarge [decay = 0.1/10 // factor = 0.3 // epochs = 25]

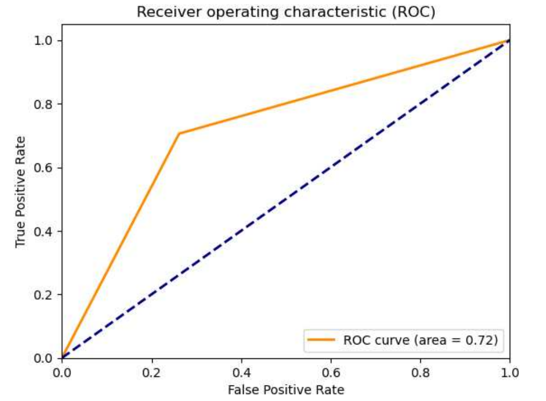

Carrillo-Larco RM, *et al. BMJ Open* 2022; 12:e063411. doi: 10.1136/bmjopen-2022-063411
