# Supplementary material for: Street images classification according to COVID-19 risk in Lima, Peru: a convolutional neural networks feasibility analysis
Source: BMJ Open. 2022 Sep 19;12(9):e063411. doi: 10.1136/bmjopen-2022-063411 (PMC9485648; doi:10.1136/bmjopen-2022-063411)
Supplement: Author's manuscript [file bmjopen-2022-063411.draft_revisions.pdf]

BMJ Open is committed to open peer review. As part of this commitment we make the peer review history of every article we publish publicly available.

When an article is published we post the peer reviewers' comments and the authors' responses online. We also post the versions of the paper that were used during peer review. These are the versions that the peer review comments apply to.

The versions of the paper that follow are the versions that were submitted during the peer review process. They are not the versions of record or the final published versions. They should not be cited or distributed as the published version of this manuscript.

BMJ Open is an open access journal and the full, final, typeset and author-corrected version of record of the manuscript is available on our site with no access controls, subscription charges or pay-per-view fees (http://bmjopen.bmj.com).

If you have any questions on BMJ Open's open peer review process please email info.bmjopen@bmj.com

BMJ Open

Street images classification according to COVID-19 risk in Lima, Peru: A convolutional neural networks feasibility analysis

Journal:	*BMJ Open*
Manuscript ID	bmjopen-2022-063411
Article Type:	Original research
Date Submitted by the Author:	30-Mar-2022

Complete List of Authors:	Carrillo-Larco, Rodrigo; Imperial College London,<br>Castillo-Cara, Manuel; Universidad Politécnica de Madrid, Ontology Engineering Group<br>Hernández Santa Cruz, Jose Francisco; Independent Researcher

Keywords:	COVID-19, EPIDEMIOLOGY, PUBLIC HEALTH

SCHOLARONE™
Manuscripts

I, the Submitting Author has the right to grant and does grant on behalf of all authors of the Work (as defined in the below author licence), an exclusive licence and/or a non-exclusive licence for contributions from authors who are: i) UK Crown employees; ii) where BMJ has agreed a CC-BY licence shall apply, and/or iii) in accordance with the terms applicable for US Federal Government officers or employees acting as part of their official duties; on a worldwide, perpetual, irrevocable, royalty-free basis to BMJ Publishing Group Ltd ("BMJ") its licensees and where the relevant Journal is co-owned by BMJ to the co-owners of the Journal, to publish the Work in this journal and any other BMJ products and to exploit all rights, as set out in our licence.

The Submitting Author accepts and understands that any supply made under these terms is made by BMJ to the Submitting Author unless you are acting as an employee on behalf of your employer or a postgraduate student of an affiliated institution which is paying any applicable article publishing charge ("APC") for Open Access articles. Where the Submitting Author wishes to make the Work available on an Open Access basis (and intends to pay the relevant APC), the terms of reuse of such Open Access shall be governed by a Creative Commons licence – details of these licences and which Creative Commons licence will apply to this Work are set out in our licence referred to above.

Other than as permitted in any relevant BMJ Author's Self Archiving Policies, I confirm this Work has not been accepted for publication elsewhere, is not being considered for publication elsewhere and does not duplicate material already published. I confirm all authors consent to publication of this Work and authorise the granting of this licence.

For peer review only

Street images classification according to COVID-19 risk in Lima, Peru: A convolutional neural networks feasibility analysis

Rodrigo M Carrillo-Larco[1,2,3]

Manuel Castillo-Cara[4,5]

Jose Francisco Hernández Santa Cruz[6]

1. Department of Epidemiology and Biostatistics, School of Public Health, Imperial College London, London, UK
2. CRONICAS Centre of Excellence in Chronic Diseases, Universidad Peruana Cayetano Heredia, Lima, Peru
3. Universidad Continental, Lima, Peru
4. Ontology Engineering Group, Universidad Politécnica de Madrid, Madrid, Spain
5. Universidad de Lima, Lima, Peru
6. Independent researcher, Edinburgh, UK

Corresponding author:

Rodrigo M Carrillo-Larco, MD

Department of Epidemiology and Biostatistics

School of Public Health

Imperial College London

rcarrill@imperial.ac.uk

For peer review only

For peer review only - http://bmjopen.bmj.com/site/about/guidelines.xhtml

ABSTRACT

Objectives: During the COVID-19 pandemic convolutional neural networks (CNNs) have been used in clinical medicine (e.g., chest X-rays classification). Whether CNNs could inform the epidemiology of COVID-19 classifying street images according to COVID-19 risk is unknown, yet it could pinpoint high-risk places and relevant features of the built environment. In a feasibility study, we trained CNNs to classify the area surrounding bus stops (Lima, Peru) into *moderate* or *extreme* COVID-19 risk.

Methods: We used five images per bus stop. The outcome label (*moderate* or *extreme*) for each bus stop was extracted from the local transport authority. We used transfer learning and updated the output layer of five CNNs: NASNetLarge, InceptionResNetV2, Xception, ResNet152V2, and ResNet101V2. We chose the best performing network which was further tuned. We used GradCam to understand the classification process.

Results: NASNetLarge outperformed the other CNNs except in the recall metric for the *moderate* label and in the precision metric for the *extreme* label; the ResNet152V2 performed better in these two metrics (85% vs 76% and 63% vs 60%, respectively). The NASNetLarge was further tuned. The best recall (75%) and F1 score (65%) for the *extreme* label were reached with data augmentation techniques. Areas close to buildings or with people were often classified as extreme risk.

Conclusions: This feasibility study showed that CNNs has the potential to classify street images according to levels of COVID-19 risk. In addition to applications in clinical medicine, CNNs and street images could advance the epidemiology of COVID-19 at the population level.

Key words: machine learning; deep learning; artificial intelligence; computer vision; built environment; population health.

For peer review only - http://bmjopen.bmj.com/site/about/guidelines.xhtml

Strengths and limitations of this study

- We used five images per bus stop and the outcome information was provided by an official Government institution.
- We leveraged on five well-known convolutional neural networks (transfer learning).
- The analysis focused on street images from one city only.
- Original data (street images) cannot be shared with the paper because of restricted access.

For peer review only

For peer review only - http://bmjopen.bmj.com/site/about/guidelines.xhtml

INTRODUCTION

In COVID-19 research, deep learning tools applied to image analysis (i.e., computer vision) have informed the diagnosis and prognosis of patients through classification of chest X-ray and computer tomography images.[1-3] These tools have helped practitioners treating COVID-19 patients.

On the other hand, the application of computer vision to study the epidemiology of COVID-19 has been limited. One relevant example is the use of Google Street View images to extract features of the built environment and associate these with COVID-19 cases in the United States.[4] This work showed that unstructured and non-conventional data sources, such as street images, can deliver relevant information to characterize the epidemiology of COVID-19 at the population level.[4] However, computer vision models to classify street images based on their COVID-19 risk do not exist. These models could be relevant to understand unique local features of the built environment related to high COVID-19 risk. In addition, these models could be applied to places where observed data are not available to identify whether this place is at moderate or high risk of COVID-19, to inform relevant interventions. This could be particularly helpful in Low- and Middle-income countries where limited resources do not allow massive COVID-19 testing, leaving places with no observed information about the COVID-19 epidemiology.

In this pilot feasibility study, we aimed to ascertain whether a convolutional neural network (deep learning) model could classify street images of bus stops according to their COVID-19 risk (binary outcome: *moderate* versus *extreme* risk) in Lima, Peru. We also aimed to understand what features of the images were most influential in the classification process. This is a pilot proof-of-concept empirical analysis.

METHODS

Study design

We used convolutional neural networks to study street images of bus stops and their surroundings in Lima, Peru. We implemented a classification model to classify the bus stops into two labels: *moderate* or *extreme* risk of COVID-19. We addressed a classification problem.

For peer review only - http://bmjopen.bmj.com/site/about/guidelines.xhtml

Rationale

We used five images per bus stop covering 360 degrees around the bus stop. Therefore, we targeted the bus stop and the surrounding area. We did not target the bust stop itself alone. The bus stop was the anchor for the outcome label (*moderate* or *extreme* risk of COVID-19) in the immediate surrounding area. It is unlikely that COVID-19 risk would be confined to the bus stop itself. Rather, the bus stop would be a proxy of the risk in the immediate nearby area.

We combined the five images before randomly splitting into the train, test and validation dataset. We used the function *train_test_split* which randomly splits the data with equal distribution of the target outcome. We did not condition the random split on the bus stops because we did not target the bus stop itself only. A random split would provide data to have different profiles of the built environment, green areas, bus stops, and other street features relevant for the model to learn and classify according to COVID-19 risk.

We deemed this a pilot feasibility proof-of-concept study because we aimed to provide preliminary data on whether convolutional neural networks could classify street images according to COVID-19 risk. There is abundant evidence about convolutional neural networks being used for classification of X-rays and other clinical images. However, there is no evidence on convolutional neural networks being used for population health. Ours is a proof-of-concept work, and future research could leverage on this idea with more images, classifying into multiple outcome labels, and implementing more sophisticated networks.

Data sources

The labels (observed data) of the bus stops were downloaded from the website of the Authority for Urban Transport in Lima and Callao (*Autoridad de Transporte Urbano para Lima y Callao* (ATU), name in Spanish). This government office manages the public transportation service in Lima, and publishes a classification map in which all bus stops in Lima are set into four categories of COVID-19 risk: moderate < high < very high < extreme.[5] Further details of their classification system have not been released. In this pilot feasibility study, we only worked with the bus stops deemed as *moderate* (label 0) and *extreme* (label 1) risk of COVID-19. We used the classification profile released on 2021-05-24.[6] We conducted a pilot feasibility study considering two outcome labels only, because we aimed to ascertain whether our hypothesis was possible and lead to relevant results. Our feasibility proof-of-concept study could demonstrate that convolutional

For peer review only - http://bmjopen.bmj.com/site/about/guidelines.xhtml

neural networks could successfully classify street images according to COVID-19 risk. This has not been studied before. Future work will leverage on this preliminary experience to develop a four-outcome model, using larger datasets and incorporating more sophisticated networks.

We used the location (longitude and latitude coordinates) of the bus stops to download their street images through the application programming interface (API) of Google Street View. That is, we downloaded all the images in one batch through the API, rather than each one at the time through the API or from the standard Google Street View website. For each bus stop (i.e., from each coordinate), we downloaded five images: when the camera was facing at 0 degrees, at 90 degrees, at 180 degrees and at 270 degrees; in addition, we also downloaded one image in which the direction of the camera was not specified (i.e., the heading parameter in the API request was set at default). In other words, for each bus stop we had five images. We did this to maximise the available data and to cover the surrounding area of the bus stop.[7] Our rationale was that the bus stop itself would not be responsible for the classification (moderate or extreme risk), but the whole nearby environment.

Original dataset

Overall, after downloading both the labels and the images, there were 1,788 bus stops with their corresponding label: 1,173 in the *moderate* category and 615 in the *extreme* category (1,173 + 615 = 1,788). Because we used five images per bus stop, the analysis included 8,940 (1,788 x 5) images and their corresponding label. The training dataset included a random sample of 60% (5,364) of the original dataset. As further explained in the next section (Data preparation and class imbalance), after correcting for class imbalance by introducing duplicates of the class with fewer observations, the training data included 7,024 observations (3,519 for *moderate* and 3,505 for *extreme* labels). The validation and test datasets included a random sample of 20% of the original dataset each (0.20 x 8,940 = 1,788); the validation and test datasets were not corrected for class imbalance.

Data preparation and class imbalance

We combined the images and the labels in one dataset, which was further divided into three datasets: the training dataset including 60% of the data, the validation dataset including 20%, and the test dataset including the remaining 20%. Data allocation to each of these three datasets was at random. After splitting

For peer review only - http://bmjopen.bmj.com/site/about/guidelines.xhtml

the data, we corrected for class imbalance in the train dataset only. We randomly multiplied the number of images in the imbalanced outcome by 0.9. This led to virtually the same number of images for the *moderate* and *extreme* risks labels.

There were two outcomes of interest: *moderate* and *extreme* risk. However, there were more observations in the *moderate* category than in the *extreme* category. That is, there was class imbalance. After splitting the data into the training, test and validation sets, we corrected for class imbalance in the training dataset only. We randomly increased the number of observations in the extreme category by 90% in the training dataset (not in validation and test datasets). The original (before correction for class imbalance) training set had 3,519 observations in the *moderate* category and 1,845 in the *extreme* category (3,519 + 1,845 = 5,346). After correcting for class imbalance as described before, the training dataset had 3,519 observations in the *moderate* category (this number did not change) and 3,505 (1.9 x 1,845) observations in the *extreme* category. Therefore, there were 3,519 (*moderate*) + 3,505 (*extreme* after class imbalance correction) = 7,024 images and labels in total in the training dataset.

Analysis

In-depth details about the analysis are available in Supplementary Materials pp. 03-06. The analysis code (Python Jupyter notebooks) is also available as Supplementary Materials.

In brief, in a pre-specified protocol we decided to elaborate on five deep convolutional neural networks pre-trained with ImageNet (i.e., transfer learning). We chose these five networks because they have the best top-5 accuracy of all models available in the Keras library:[8] NASNetLarge, InceptionResNetV2, Xception, ResNet152V2 and ResNet101V2. We implemented these five models with the same hyper-parameters, and then we selected the one with the best performance which was further tuned and tested. The image classification model was based on the latter model only (i.e., the one with the best performance out of the five candidate models). We reported the loss and accuracy in the validation and test datasets; we also used the test dataset to report the accuracy, recall and F1 score for each of the two possible outcomes (*moderate* or *extreme* risk). Finally, we used GradCam to identify which areas of the input image were more relevant to inform the classification process;[9] for this, we randomly selected four images per outcome (i.e., four images from the *moderate* label and four images from the *extreme* label).

For peer review only - http://bmjopen.bmj.com/site/about/guidelines.xhtml

Ethical statement

Human subjects were not directly studied in this work. The analysed data are in the public domain and can be downloaded. The analysed data do not contain any personal identifiers. We did not seek approval by an ethics committee.

Patient and public involvement

Human subjects did not participate nor were involved in this study.

RESULTS

Selection of the pre-trained model out of five candidate models

We used transfer learning and updated the output layer of five convolutional neural networks to predict our two classes of interest. The NASNetLarge architecture and weights outperformed the other candidate convolutional neural networks, except in the recall metric for the *moderate* label: 76% versus 85% in NASNetLarge and ResNet152V2, respectively (Table 1). The ResNet152V2 also performed better than the NASNetLarge in the precision metric for the *extreme* label (60% vs 63%). Further experiments were only conducted with NASNetLarge because, overall, it performed better than the other pre-trained networks.

Model performance

We further tuned NASNetLarge with different hyper-parameters aiming to improve the accuracy (Table 2).

First, building on the initial hyper-parameters, we implemented two data augmentation options: horizontal flip and zoom range. We chose these two data augmentation methods because they appropriately fit the images under analysis; for example, because we were working with street images, a vertical flip would not seem appropriate. The new model with horizontal flip improved the recall and F1 score for the *extreme* label; from 68% with the original NASNetLarge to 75%, and from 64% to 65% (Figure 1). The new model with horizontal flip and zoom range at 30% had better performance that the original NASNetLarge model in six out of ten parameters, including precision for the *extreme* label.

Second, also building on the initial hyper-parameters (i.e., without data augmentation), the decay in the stochastic gradient descendent optimizer was changed from 1/25 (25 was the number of epochs) to 1/10

For peer review only - http://bmjopen.bmj.com/site/about/guidelines.xhtml

(the number of epochs was not changed). This model did not substantially improve the performance of the model.

Third, building on the last specification (i.e., model with a decay of 1/10), we updated the monitoring factor which updated the learning rate when it did not improve through epochs. Originally, this factor was 0.1, and we updated it to 0.3. This model did not substantially improve the performance of the model.

GradCam

In the GradCam analysis we used the NASNetLarge model with one data augmentation technique (horizontal flip). Even though the performance of the NASNetLarge model with two data augmentation techniques (horizontal flip and zoom range) was better in more metrics, the model with horizontal flip only had better recall and F1 score for the *extreme* label. The main indications for a *moderate* risk classification were the presence of green areas and lack of close nearby buildings (Figure 2). Areas close to buildings or with a considerable presence of people were often classified as extreme COVID-19 risk. The presence of cars did not seem to impact the classification process.

DISCUSSION

Main findings

With almost all research on computer vision and COVID-19 focusing on diagnostic models based on X-rays and other clinical images, our proof-of-concept work is novel because it borrows techniques from computer vision into epidemiology and population health leveraging on available data (street images). This is the first work of this kind, to the best of our knowledge. In this pilot feasibility proof-of-concept study, we showed that deep convolutional neural networks can classify street images according to their COVID-19 risk with acceptable accuracy. Future work should strengthen available convolutional neural networks or develop a new architecture which could maximize the accuracy classification, not only for a binary outcome but also covering multiple outcomes. This work could spark interest to use convolutional neural networks –and other artificial intelligence tools– to advance population health and the epidemiological knowledge of COVID-19 (and other diseases), above and beyond the applications of convolutional neural networks for diagnosis and prognosis of individual patients (e.g., classification of chest X-rays and compute tomography images[1-3]).

For peer review only - http://bmjopen.bmj.com/site/about/guidelines.xhtml

Results in context

This feasibility proof-of-concept work signalled that a deep neural network is moderately accurate to classify street images according to COVID-19 risk levels. These results are encouraging because the task we pursued was difficult: to classify street images into levels for which there is no unique intrinsic information in the images. Classification of, for example, chest X-ray images into healthy or ill could be easier for a convolutional neural network because the X-ray of someone with a disease (e.g., pneumonia) would have unique features (e.g., infiltrate spots at the bottom of the lungs) that a chest X-ray of someone healthy would not have at all. Conversely, in our case, the street images did not have a unique underlying pattern to guide the classification process. Our model had to work harder to find those unique characteristics to decide between *moderate* and *extreme* risk.

Further tuning of the selected model (NASNetLarge) suggested that data augmentation methods improved the performance of the model. When we updated the learning rate optimizer (decay and factor parameters), the model performance did not substantially improve. This could suggest that for this particular task we may need a large number of images. Alternatively, several combinations of data augmentation techniques would need to be tested. Data augmentations should be carefully considered to select those most suitable for these images; for example, vertical flip may not be a reasonable choice for street images.

Nguyen and colleagues used Google Street View images to associate features of the built environment with COVID-19 cases in several states in the United States.[4] Although we could have followed the same approach, there would be some unique local features of the built environment that may not have been identified by available object detection tools (e.g., street vendors and newspaper stands). We are not aware of other peer-reviewed papers in which street images have been classified according to COVID-19 outcomes. Our work contributes to the available literature with a newly trained model benefiting from transfer learning from a large and well-known architecture (NASNetLarge), based on images from a city in an upper-middle income country (Lima, Peru).

The activation maps (GradCam) are not only useful to analyse the model's interpretation capability, but they bolster the existing evidence of crowded places or indoor venues (such as nearby buildings) as COVID-19 high-risk areas; on the other hand, open spaces, such as green areas or locations far from buildings, remain

For peer review only - http://bmjopen.bmj.com/site/about/guidelines.xhtml

For peer review only

as moderate or low COVID-19 risk areas. Overall, our findings agree with the evidence describing crowded areas, such as restaurants, gyms, hotels, and cafes, as having high COVID-19 transmission risk.[10]

Public health implications

Our work could have pragmatic applications to better understand the epidemiology of COVID-19 and to inform public health measures. For example, our model –and future work improving this analysis– could be used to characterize bus stops and other public places for which labelled data are not available. We worked with images from bus stops in Lima, and our model could be applied to bus stops in other cities to characterize their COVID-19 risk. Furthermore, our work could spark interest to conduct more sophisticated analyses, like semantic segmentation whereby some unique elements of the local environment could be identified as potential high-risk places. For example, bus stops often host food street vendors and newspaper stands where people usually gather. Perhaps, the bus stops themselves are not high-risk places, but the surrounding shops. This could inform policies and interventions to reduce the COVID-19 risk in these places. Overall, deep learning techniques, including convolutional neural networks, could be adopted by epidemiological research to advance the evidence about risk factors as well as disease outcomes and distribution, in addition to their current use in clinical medicine.[1-3]

As argued in the introduction, this is a pilot feasibility proof-of-concept study to study whether convolutional neural networks could classify street images according to COVID-19 indicators. This work complements the current use of convolutional neural networks for COVID-19 classification of clinical images (e.g., X-rays). This work should be regarded as the first step in the use of convolutional neural networks in epidemiology and population health relevant to COVID-19; this work is not the ultimate work on this subject and future research should improve our approach and results.

Ongoing and future work

Ongoing and future work includes the development of a classification model for the four outcome labels (i.e., *moderate*, *high*, *very high* and *extreme* COVID-19 risk). We will implement techniques that can potentiate the classification capacity of the neural networks, including ensemble models,[11] novel loss functions not currently implemented in the Keras environment (e.g., squared earth mover's distance-based loss function),[12] and we may try other architectures (e.g., SqueezeNet[13]) with similar precision yet less

For peer review only - http://bmjopen.bmj.com/site/about/guidelines.xhtml

computationally expensive. Because most of our bus stop images also depicted buildings, we may try to use a network already trained on images of buildings and other city landscapes (e.g., Places-365).

Strengths and limitations

In this preliminary proof-of-concept work, we followed a pre-defined protocol which included transfer learning leveraging on large and deep neural networks trained with millions of images (ImageNet). We still had to train the parameters of the output layer, for which we did not have a massive number of images. Future work could expand our analysis with information and images from more bus stops or other public spaces to train a more robust model. Ideally, these images should come from different cities. This information may be available in other countries. There are further limitations we must acknowledge. First, the images and labels were not synchronic; that is, the figures and the labels were not collected on the same date. This is a shared limitation with other studies working with street images from open sources (e.g., Google Street View), because these images are not taken continuously or in real time. This should not be a major limitation because the analysis mostly focused on the built environment which has not changed substantially in recent years. Because this feasibility study showed that the classification model performed moderately well, researchers could collect new images in a prospective work to verify our findings with synchronic data. In this line, satellite images collected daily could be useful. Second, we did not have exact details on how the bus stops were classified by the local authorities. Nevertheless, we used official information which is provided to the public for their safety and to inform them about the progression of the COVID-19 pandemic in Lima, Peru. Therefore, we trust their method for classification is sound and based on the best available evidence. Third, we had five images per bus stop: the fifth image did not look at a specific angle, unlike the other four images that looked at 0, 90, 180 and 270 degrees around the bus stop. Therefore, the fifth image had some overlap with the other images. We took this decision to maximize the available data. Researchers with access to more labelled information, perhaps from public places overseas, could use the four images without overlap and not significantly reducing the dataset size. In this line, the datasets (training, test and validation) were split randomly and, just by chance albeit improbably, all images of one particular bus stop could have fallen in a subset (e.g., test dataset). If so, the model would have poor accuracy to predict this specific bus stop because the model did not have any information/images about that bus stop in the training dataset. However, because we trained the model to classify *moderate* and *extreme* risk of COVID-19, the

For peer review only - http://bmjopen.bmj.com/site/about/guidelines.xhtml

289 model learnt patterns and profiles of the bus stops and their surrounding areas. This training could then be
290 applied to other bus stops with similar characteristics. The GradCam analysis helped us to exemplify the
291 patterns most influential in the selection process. Arguably, the influential patterns would be in all or most
292 images. Fourth, our model cannot be independently reproduced because we could not make the underlying
293 data available because these images do not belong to us. Google Street View images are available through
294 the API, though they need personal login credentials. Although this would not replace the raw underlying
295 data, to increase the transparency of our work we made available the Jupyter notebooks used in the analysis
296 (Supplementary Materials). These notebooks show the codes and results. Fifth, we did not report or discuss
297 the algorithms or computations behind the convolutional neural networks we used for transfer learning. As
298 per our protocol, we chose and applied a set of established convolutional neural networks to solve a
299 classification problem. Disentangling the underlying mechanisms underneath each convolutional neural
300 network was beyond the scope of this work. Nevertheless, it is relevant to understand the areas of the
301 images most influential in the classification process. This way we can verify if the classification process
302 followed a logical path. We therefore presented the GradCam analysis.

303 **Conclusions**

304 This pilot feasibility proof-of-concept study showed that a convolutional neural network has moderate
305 accuracy to classify street images into *moderate* and *extreme* risk of COVID-19. In addition to applications
306 in clinical medicine, deep convolutional neural networks have the potential to also advance the epidemiology
307 of COVID-19 at the population level exploding unstructured and non-conventional data sources.

For peer review only - http://bmjopen.bmj.com/site/about/guidelines.xhtml

TABLES

Table 1: Performance of the five candidate convolutional neural networks

	NASNetLarge	InceptionResNetV2	Xception	ResNet152V2	ResNet101V2
**Loss, validation**	0.526799	0.554040	0.533278	0.793147	0.744385
**Accuracy, validation**	0.742046	0.713636	0.730682	0.721023	0.723295
**Loss, test**	0.539906	0.557637	0.555917	0.800661	0.726274
**Accuracy, test**	0.731818	0.721591	0.706818	0.722727	0.718750
**Precision, label 0 (*moderate*)**	0.82	0.78	0.82	0.76	0.80
**Recall, label 0 (*moderate*)**	0.76	0.81	0.71	0.85	0.76
**F1 score, label 0 (*moderate*)**	0.79	0.79	0.76	0.80	0.78
**Precision, label 1 (*extreme*)**	0.60	0.61	0.56	0.63	0.58
**Recall, label 1 (*extreme*)**	0.68	0.56	0.70	0.48	0.64
**F1 score, label 1 (*extreme*)**	0.64	0.58	0.62	0.54	0.61

Green colour highlights the best metric, yellow colour highlights the second best metric and red colour highlights the third best metric row-wise. The precision, recall and F1 score are presented as proportions (multiply by 100 to have percentages). The precision, recall and F1 score were computed with the test dataset. Receiver Operating Characteristic (ROC) Curves for each model are available in Supplementary Materials.

For peer review only - http://bmjopen.bmj.com/site/about/guidelines.xhtml

Table 2: Further tuning of the selected model (NASNetLarge) and the performance metrics

		New model specifications			
	**Original model (as in Table1)**	**horizontal_flip = True // epochs = 25 (stopped at 12)**	**horizontal_flip = True // zoom_range = 0.30 // epochs = 25 (stopped at 15)**	**decay = 0.1/10 // epochs = 25 (stopped at 12)**	**decay = 0.1/10 // factor = 0.3 // epochs = 25 (stopped at 12)**
**Loss, validation**	0.526799	0.534797	0.537553	0.532246	0.532246
**Accuracy, validation**	0.742046	0.737500	0.739773	0.732386	0.732386
**Loss, test**	0.539906	0.550286	0.528204	0.538252	0.538252
**Accuracy, test**	0.731818	0.719318	0.735795	0.725568	0.725568
**Precision, label 0 (*moderate*)**	0.82	0.85	0.76	0.83	0.83
**Recall, label 0 (*moderate*)**	0.76	0.71	0.87	0.74	0.74
**F1 score, label 0 (*moderate*)**	0.79	0.77	0.81	0.78	0.78
**Precision, label 1 (*extreme*)**	0.60	0.57	0.66	0.59	0.59
**Recall, label 1 (*extreme*)**	0.68	0.75	0.47	0.71	0.71
**F1 score, label 1 (*extreme*)**	0.64	0.65	0.55	0.64	0.64

Green colour highlights the best metric, yellow colour highlights the second best metric and red colour highlights the third best metric row-wise considering only the new model specifications. The precision, recall and F1 score are presented as proportions (multiply by 100 to have percentages). Receiver Operating Characteristic (ROC) Curves for each model are available in Supplementary Materials.

For peer review only - http://bmjopen.bmj.com/site/about/guidelines.xhtml

FIGURES

Figure 1: Confusion matrix for the best NASNetLarge model

This NASNetLarge model corresponds to the one with data augmentation of horizontal flip (first column in the new model specification section of Table 2). The figure shows the absolute number of images in each label: observed (true) on the y-axis and predicted on the x-axis.

Figure 2: GradCam representation of four randomly selected images per outcome label (moderate risk on the left and extreme risk on the right) and based on the final model

Final model corresponds to the NASNetLarge showed in the first column in the new model specification section of Table 2. The four images per outcome label were randomly chosen. From green to red, it shows the areas that influenced the most in the classification process; that is, areas in red were the most influential in the classification process. Images on the left column belong to the moderate label and images on the right column belong to the extreme label. Images courtesy of Google Maps View.

For peer review only

For peer review only - http://bmjopen.bmj.com/site/about/guidelines.xhtml

CONTRIBUTIONS

RMC-L and JFHSC conceived the idea. RMC-L conducted the analysis with support from JFHSC and MC-C. MC-C supported the revised analysis. All authors approved the submitted version.

FUNDING

RMC-L is supported by a Wellcome Trust International Training Fellowship (Wellcome Trust 214185/Z/18/Z).

COMPETING INTERESTS

No conflict of interest.

DATA AVAILABILITY STATEMENT

Outcome (i.e., labels: moderate and extreme COVID-19 risk) data are available online: https://sistemas.atu.gob.pe/paraderosCOVID; this information was systematized at https://github.com/jmcastagnetto/lima-atu-covid19-paraderos. The images were downloaded from Google Street View through the API with a personal account; images cannot be shared with third parties. All analysis codes are available as Python Jupyter Notebooks in Supplementary Materials. JupyterLab Notebooks and the final model (weights) are available at: https://figshare.com/articles/online_resource/Street_images_classification_according_to_COVID-19_risk_in_Lima_Peru_A_convolutional_neural_networks_feasibility_analysis/17321021

For peer review only

REFERENCES

1. Ghaderzadeh M, Asadi F. Deep Learning in the Detection and Diagnosis of COVID-19 Using Radiology Modalities: A Systematic Review. *Journal of healthcare engineering* 2021; **2021**: 6677314.

2. Mohammad-Rahimi H, Nadimi M, Ghalyanchi-Langeroudi A, Taheri M, Ghafouri-Fard S. Application of Machine Learning in Diagnosis of COVID-19 Through X-Ray and CT Images: A Scoping Review. *Frontiers in cardiovascular medicine* 2021; **8**: 638011.

3. Montazeri M, ZahediNasab R, Farahani A, Mohseni H, Ghasemian F. Machine Learning Models for Image-Based Diagnosis and Prognosis of COVID-19: Systematic Review. 2021; **9**(4): e25181.

4. Nguyen QC, Huang Y, Kumar A, et al. Using 164 Million Google Street View Images to Derive Built Environment Predictors of COVID-19 Cases. 2020; **17**(17).

5. Autoridad de Transporte Urbano para Lima y Callao (ATU). Paraderos con Riesgo de COVID - 19. URL: https://sistemas.atu.gob.pe/paraderosCOVID.

6. URL: https://github.com/jmcastagnetto/lima-atu-covid19-paraderos.

7. Suel E, Polak JW, Bennett JE, Ezzati M. Measuring social, environmental and health inequalities using deep learning and street imagery. *Scientific reports* 2019; **9**(1): 6229.

8. Keras applications. URL: https://keras.io/api/applications/.

9. Selvaraju RR, Cogswell M, Das A, Vedantam R, Parikh D, Batra D. Grad-CAM: Visual Explanations from Deep Networks via Gradient-Based Localization. *International Journal of Computer Vision* 2020; **128**(2): 336-59.

10. Chang S, Pierson E, Koh PW, et al. Mobility network models of COVID-19 explain inequities and inform reopening. *Nature* 2021; **589**(7840): 82-7.

11. Ganaie M, Hu M. Ensemble deep learning: A review. *arXiv preprint arXiv:210402395* 2021.

12. Hou L, Yu C-P, Samaras D. Squared earth mover's distance-based loss for training deep neural networks. *arXiv preprint arXiv:161105916* 2016.

13. Iandola FN, Han S, Moskewicz MW, Ashraf K, Dally WJ, Keutzer K. SqueezeNet: AlexNet-level accuracy with 50x fewer parameters and< 0.5 MB model size. *arXiv preprint arXiv:160207360* 2016.

For peer review only

For peer review only - http://bmjopen.bmj.com/site/about/guidelines.xhtml

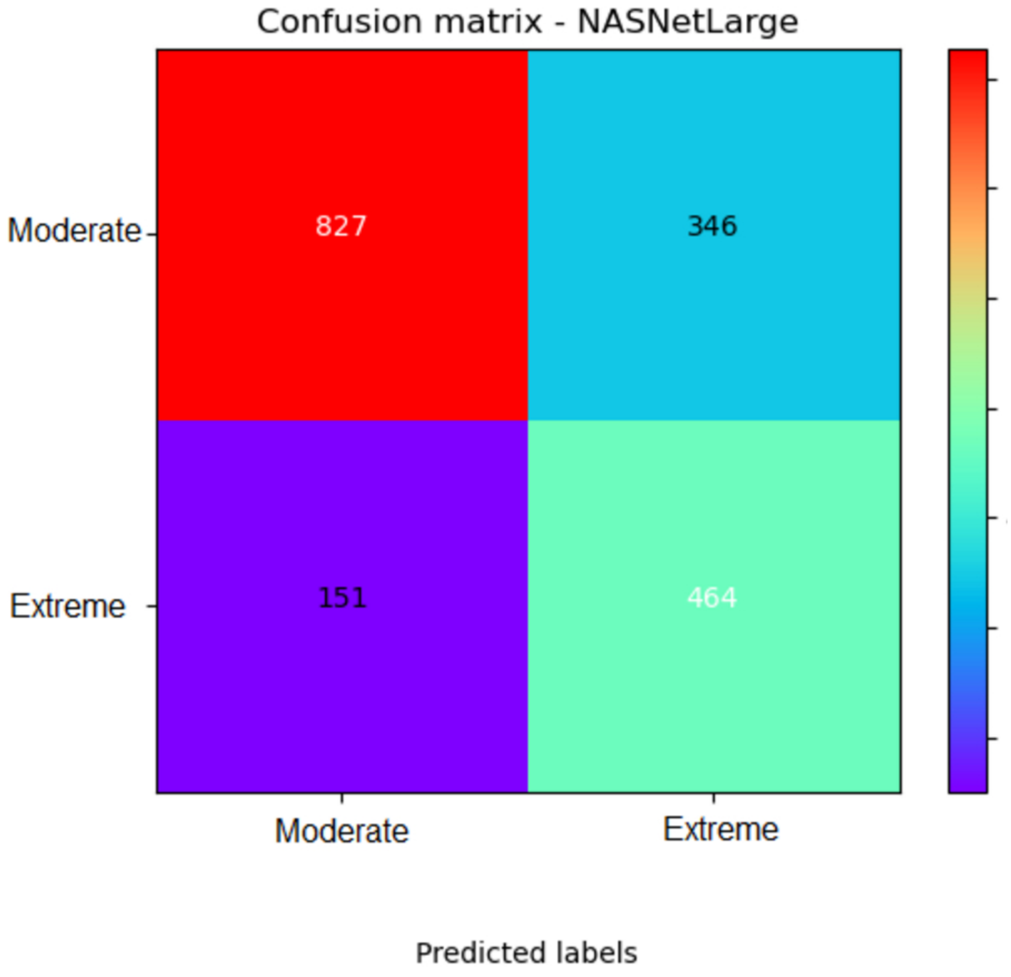

Figure 1: Confusion matrix for the best NASNetLarge model

This NASNetLarge model corresponds to the one with data augmentation of horizontal flip (first column in the new model specification section of Table 2). The figure shows the absolute number of images in each label: observed (true) on the y-axis and predicted on the x-axis.

601x601mm (38 x 38 DPI)

For peer review only - http://bmjopen.bmj.com/site/about/guidelines.xhtml

Supplementary Materials

Street images classification according to COVID-19 risk in Lima, Peru: A convolutional neural networks feasibility analysis

Corresponding author:

Rodrigo M Carrillo-Larco, MD

Department of Epidemiology and Biostatistics

School of Public Health

Imperial College London

rcarrill@ic.ac.uk

For peer review only

For peer review only - http://bmjopen.bmj.com/site/about/guidelines.xhtml

Contents

EXPANDED METHODS 3

Overview 3

System details 3

Images 3

Labels (outcome) 4

Class imbalance 4

Image data generator 4

Convolutional neural networks 5

Model Architecture 5

Model Training 6

Activation Heatmap by GradCam Visualization 7

References 7

Receiver Operating Characteristic (ROC) Curves I 9

Receiver Operating Characteristic (ROC) Curves II 10

For peer review only

EXPANDED METHODS

Overview

In a pre-specified protocol we decided to test five well-known convolutional neural network architectures. These five networks are those with the best accuracy among the models available in the Keras library.[1] From these five candidate models, we chose the one with the best performance for our task; this model was further tuned to improve its performance.

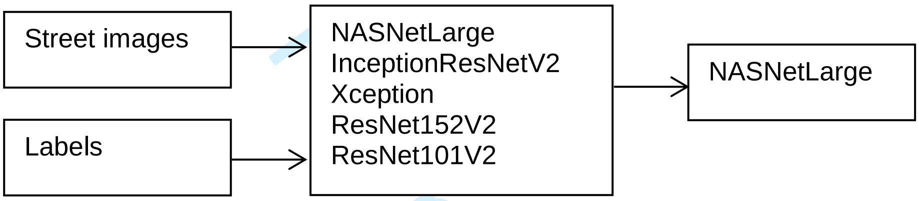

Analysis code (Jupyter notebooks) and the weights of the final model are found here: https://drive.google.com/file/d/1HXLsenn7yvxri7n2xE80WMtxQzj5fgBX/view?usp=sharing

System details

Analyses were conducted with a GPU NVIDIA Quadro P1000 on Python Jupyter (version 3.7.10). The notebooks are provided as supplementary materials.

Images

The list of bus stop in Lima, Peru, and their COVID-19 risk, are provided by local transport authority.[2] This information has been extracted and homogenized and is available online.[3] Key for our work, this information contains: i) location of each bus stop (latitude and longitude coordinates), which was used to extract street images; and ii) the COVID-19 risk level assigned to each bus stop, which was used as the outcome labels.

We downloaded the images from Google Street View through the application programming interface (API). We used the Python libraries *google_streetview.api* and *request*. For each bus stop (i.e., for each latitude and longitude coordinate), the API request specified the heading parameter = [0, 90, 180, 270]; in addition, we downloaded one image for which the heading parameter was set at default. Consequently, for each bus stop we had five images in total. The images were downloaded with size 640x640 pixels. In this feasibility study there were 1,788 bus stops, and because we had five images per bus stop, we used 8,940 (1,788 x 5) images in total.

For peer review only - http://bmjopen.bmj.com/site/about/guidelines.xhtml

Labels (outcome)

We used the bus stop classification released on 2021-05-24,[2, 3] by the local transport authority in Lima, Peru.[2] They classify the bus stops in four categories of COVID-19 risk: moderate, high, very high and extreme risk.[2] Details on how they made this classification are not available. Nevertheless, because this is official information released by a public authority to inform the general population, we trust their classification is based on the best available evidence. In this feasibility work we only used bus stops labelled as *moderate* (n=1,173) and *extreme* (n=615) COVID-19 risk. There were 1,788 (1,173 + 615) bus stops in total. The list of labels was appended five times, so that we would have as many images as labels (NB: we had five images per bus stop as described above).

Class imbalance

There were two outcomes of interest: moderate and extreme risk. However, there were more observations in the moderate category than in the extreme category. That is, there was class imbalance. After splitting the data into the training, test and validation sets, we corrected for class imbalance. We randomly increased the number of observations in the extreme category by 90% in the training dataset (not in validation and test datasets). The original (before correction for class imbalance) training set had 3,519 observations in the moderate category and 1,845 in the extreme category (3,519 + 1,845 = 5,346). After correcting for class imbalance as described before, the training dataset had 3,519 observations in the moderate category (this number did not change) and 3,505 (~1.9 x 1,845) observations in the extreme category (3,519 + 3,505 = 7,024 total sample in the training dataset).

Image data generator

We constructed a dataframe with two columns: the path to each image (i.e., to the exact location were the images were saved) and the corresponding label for each image. This dataframe was passed to the *ImageDataGenerator* function of the *keras.preprocessing.function* library. At this point, we also re-scaled the images between 0 and 1 by dividing by 255. Then, we created three image iterators: one for the training, validation and test datasets (*train_datagen.flow_from_dataframe* function). To the *train_datagen.flow_from_dataframe* function we passed the datarame with the location of the images and their labels, specified this was a categorical classification problem, a batch size of 32, and a

For peer review only - http://bmjopen.bmj.com/site/about/guidelines.xhtml

specific image size for each candidate model (see table below); in addition, the image iterator for the training dataset had the shuffle parameter as *True*.

Convolutional neural networks

We decided to train five candidate convolutional neural networks with the following specifications.

	NASNetLarge	InceptionResNetV2	Xception	ResNet152V2	ResNet101V2
**Image size**	331 x 331	299 x 299		224 x 224	
**Pre-trained weights**	ImageNet				
**Top layer included?**	No				
**Trainable parameters**	None				
**Additional layers**	Dense layer with 2 neurons (for the two outcome labels), with *softmax* activation				
**Number of epochs for training**	25				
**Optimizer**	SGD(learning_rate = 0.1, momentum = 0.9, decay = 0.1/number of epochs, nesterov = True)				
**Loss function**	Binary crossentropy				

SDG: stochastic descent gradient.

In addition, we monitored the validation loss: when the validation loss would not improve in one epoch, then the learning rate was multiplied by 0.1. We also specified an early stop: when the validation loss would not improve for ten epochs, the training would stop. To choose between the five candidate models we did not implement any data augmentation methods.

We chose the model with the best performance (Table 1 in the main text), which was further tuned (Table 2 in main text).

Model Architecture

The chosen model (NASNetLarge) presented in this article is a state-of-the-art convolutional neural network (CNN) pre-trained with the ImageNet dataset of images. The NASNetLarge neural network is widely used in computer vision. It is composed of several layers of convolutional cells that extract the most important features from each image, learning which features are the most characteristic from each image category. Most pre-trained state-of-the-art CNNs, such as the AlexNet or the ResNet50, base their innovations in the use of complex activation functions, efficient designs and special layers, such as the Batch Normalization,[4] which not only improve accuracy performance, but also reduce the time needed to train a model. The ResNet50 neural network, for example, introduces the concept of residual neural networks, layers that skip their connections to other layers by adding connection shortcuts, thus reducing backpropagation training time and better generalizing models.

For peer review only - http://bmjopen.bmj.com/site/about/guidelines.xhtml

However, most of these CNNs had complex design that was built by using trial-and-error methods, oblivious to modern state-of-the-art optimization methods such as the use of evolutionary and nature-inspired algorithms or Reinforcement Learning (RL). This is where Neural Search Architecture (NAS) Networks come in play.[5] Dubbed as a breakthrough in machine learning automation, NAS networks use *AI for AI.* The network's whole architecture is designed by optimization algorithms, such as Gradient-based search and RL. The idea behind this is that, setting the right restrictions to avoid repetitive inclusion of layers, the network cannot only be trained by its parameters, but by its own architecture, adding and changing layers, activations functions and connections.

Our research uses the NASNetLarge model available by Keras,[1] pre-trained on the ImageNet dataset on 1000 categories. Because we only have two categories to train on (moderate and extreme), we started by replacing the network's fully connected classification layer by a 2-neurons layer, with a softmax activation function.[1] The rest of the original NASNetLarge network was kept unmodified to preserve its proven architecture.

Model Training

To train our model we used transfer learning. Based on the pre-trained NASNet model, we retained its parameters and froze them, keeping just trainable the last fully connected layer, initializing its parameters randomly by He uniform initialization.[6] This single-stage training took advantage of the pre-trained parameters speeding up training by just focusing on the last decision layer. The model was trained for 30 epochs. Each epoch is understood as a complete training cycle through the whole train dataset. Data is then fed by batches; once all batches are loaded, an epoch is finished. By using a batch size of 32, training and testing sets are fed to the neural network. The loss function, as we are dealing with a binary classification model, is binary cross entropy.

As optimizer we used the Stochastic Gradient Descent (SGD).[7] One of the key advantages of this optimizer is due to its stochasticity in selecting each batch for the backpropagation step. Although the model takes longer to converge, unlike other optimizers, the SGD has been proven to converge better local optima, searching for global optima with much more easiness. This factor helps also to reduce overfitting.

For peer review only - http://bmjopen.bmj.com/site/about/guidelines.xhtml

Activation Heatmap by GradCam Visualization

Model interpretability is a feature that has gained importance since the appearance of methods such as GradCam.[8] This technique uses the values from the gradients in the model's final feature layer to produce visual explanations, highlighting regions of importance taken by the model to infer a given input. In other words, this technique informs which areas of the input figure were more relevant to make the final classification.

Regions that represent higher gradient values, accounting for most of the last layer activations and network's decision, are represented by being coloured closer to the red portion of the spectrum. By comparison, regions with the lowest activations, not adding much information to the network's final decision, appear as areas closer to the blue portion of the spectrum. These gradient values are taken from the network's last convolutional layer, right before the last pooling layer.

References

1. Keras applications. URL: https://keras.io/api/applications/.

2. Autoridad de Transporte Urbano para Lima y Callao (ATU). Paraderos con Riesgo de COVID - 19. URL: https://sistemas.atu.gob.pe/paraderosCOVID.

3. URL: https://github.com/jmcastagnetto/lima-atu-covid19-paraderos.

4. Ioffe S, Szegedy C. Batch Normalization: Accelerating Deep Network Training by Reducing Internal Covariate Shift. *arXiv e-prints* 2015: arXiv:1502.03167.

5. Kyriakides G, Margaritis K. An Introduction to Neural Architecture Search for Convolutional Networks. *arXiv e-prints* 2020: arXiv:2005.11074.

6. He K, Zhang X, Ren S, Sun J. Delving Deep into Rectifiers: Surpassing Human-Level Performance on ImageNet Classification. *arXiv e-prints* 2015: arXiv:1502.01852.

7. Loshchilov I, Hutter F. SGDR: Stochastic Gradient Descent with Warm Restarts. *arXiv e-prints* 2016: arXiv:1608.03983.

8. Selvaraju RR, Cogswell M, Das A, Vedantam R, Parikh D, Batra D. Grad-CAM: Visual Explanations from Deep Networks via Gradient-Based Localization. *International Journal of Computer Vision* 2020; **128**(2): 336-59.

For peer review only - http://bmjopen.bmj.com/site/about/guidelines.xhtml

For peer review only

For peer review only - http://bmjopen.bmj.com/site/about/guidelines.xhtml

Receiver Operating Characteristic (ROC) Curves I

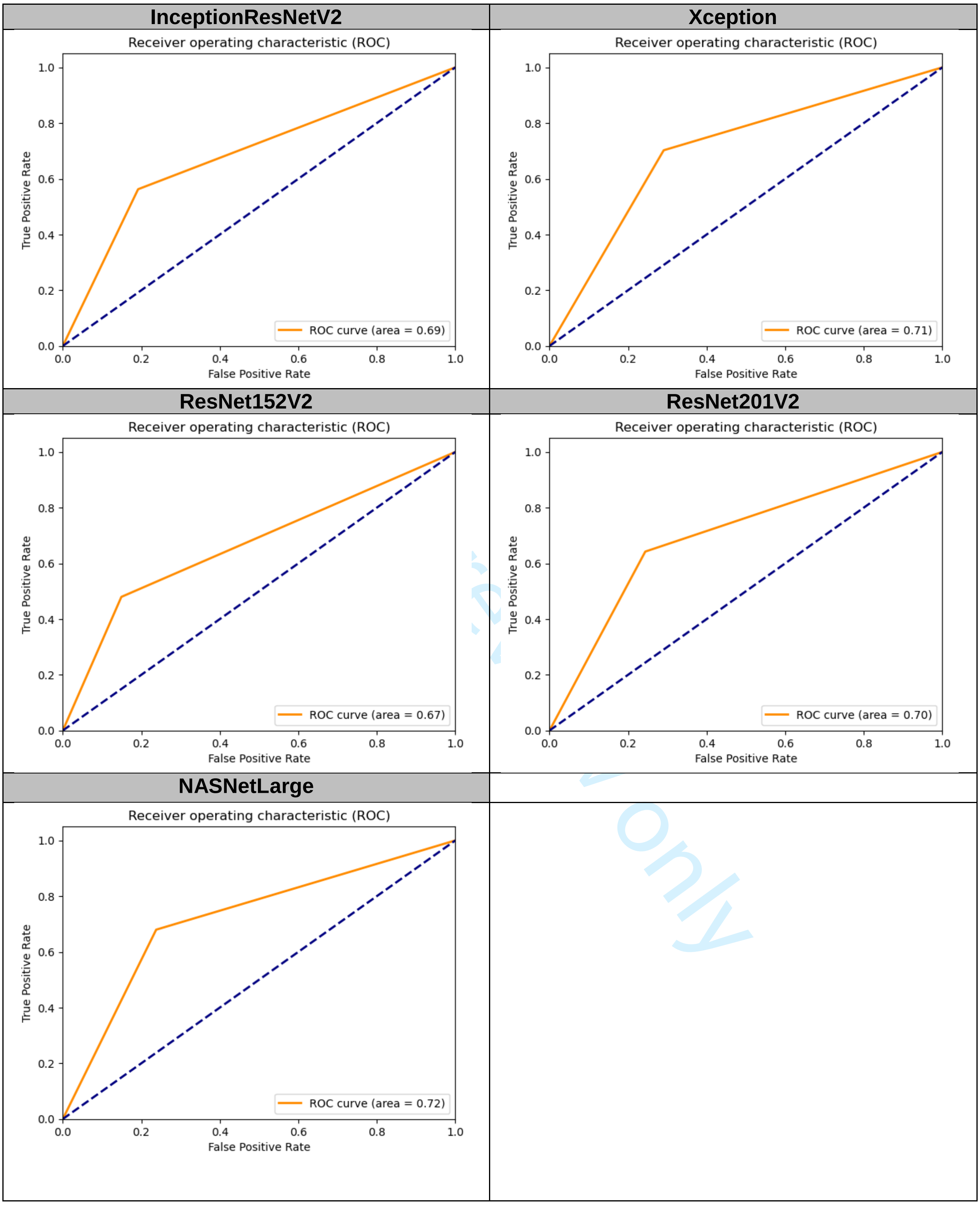

For peer review only - http://bmjopen.bmj.com/site/about/guidelines.xhtml

Receiver Operating Characteristic (ROC) Curves II

NASNetLarge [horizontal_flip = True // epochs = 25]

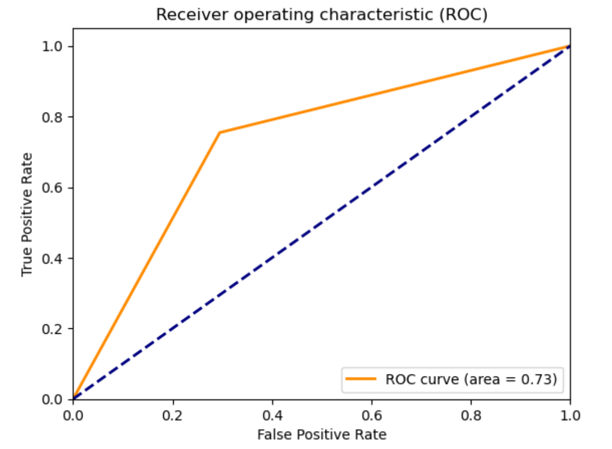

NASNetLarge [horizontal_flip = True // zoom_range = 0.30 // epochs = 25]

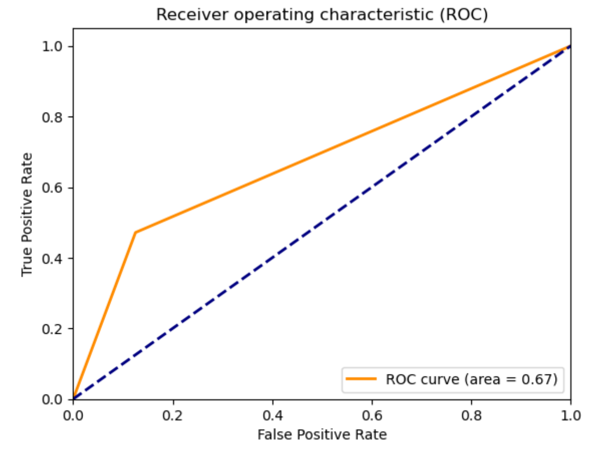

NASNetLarge [decay = 0.1/10 // epochs = 25]

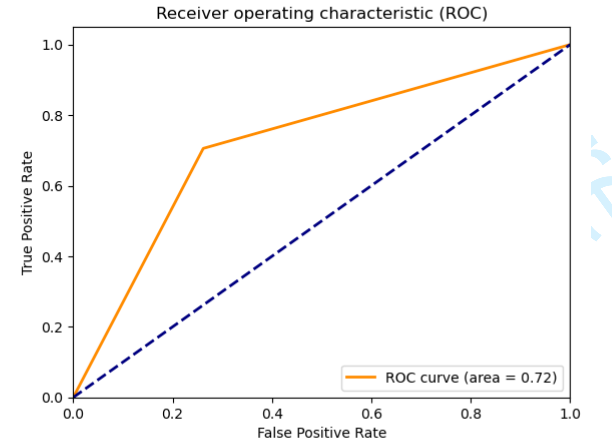

NASNetLarge [decay = 0.1/10 // factor = 0.3 // epochs = 25]

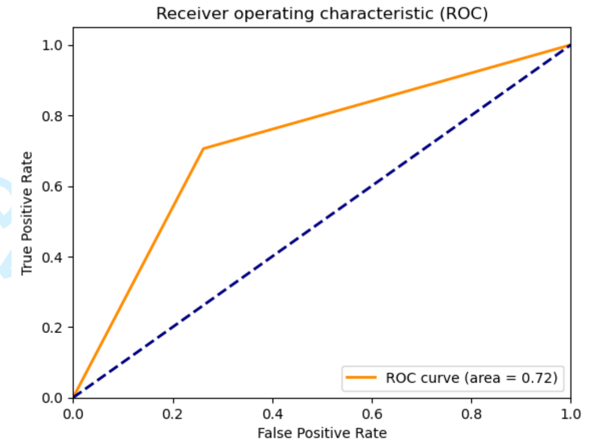

only

For peer review only - http://bmjopen.bmj.com/site/about/guidelines.xhtml

BMJ Open

Street images classification according to COVID-19 risk in Lima, Peru: A convolutional neural networks feasibility analysis

Journal:	*BMJ Open*
Manuscript ID	bmjopen-2022-063411.R1
Article Type:	Original research
Date Submitted by the Author:	27-Jul-2022
Complete List of Authors:	Carrillo-Larco, Rodrigo; Imperial College London,<br>Castillo-Cara, Manuel; Universidad Politécnica de Madrid, Ontology Engineering Group<br>Hernández Santa Cruz, Jose Francisco; Independent Researcher
**Primary Subject Heading**:	Global health
Secondary Subject Heading:	Epidemiology
Keywords:	COVID-19, EPIDEMIOLOGY, PUBLIC HEALTH

SCHOLARONE™
Manuscripts

For peer review only - http://bmjopen.bmj.com/site/about/guidelines.xhtml

I, the Submitting Author has the right to grant and does grant on behalf of all authors of the Work (as defined in the below author licence), an exclusive licence and/or a non-exclusive licence for contributions from authors who are: i) UK Crown employees; ii) where BMJ has agreed a CC-BY licence shall apply, and/or iii) in accordance with the terms applicable for US Federal Government officers or employees acting as part of their official duties; on a worldwide, perpetual, irrevocable, royalty-free basis to BMJ Publishing Group Ltd ("BMJ") its licensees and where the relevant Journal is co-owned by BMJ to the co-owners of the Journal, to publish the Work in this journal and any other BMJ products and to exploit all rights, as set out in our licence.

The Submitting Author accepts and understands that any supply made under these terms is made by BMJ to the Submitting Author unless you are acting as an employee on behalf of your employer or a postgraduate student of an affiliated institution which is paying any applicable article publishing charge ("APC") for Open Access articles. Where the Submitting Author wishes to make the Work available on an Open Access basis (and intends to pay the relevant APC), the terms of reuse of such Open Access shall be governed by a Creative Commons licence – details of these licences and which Creative Commons licence will apply to this Work are set out in our licence referred to above.

Other than as permitted in any relevant BMJ Author's Self Archiving Policies, I confirm this Work has not been accepted for publication elsewhere, is not being considered for publication elsewhere and does not duplicate material already published. I confirm all authors consent to publication of this Work and authorise the granting of this licence.

For peer review only

Street images classification according to COVID-19 risk in Lima, Peru: A convolutional neural networks feasibility analysis

Rodrigo M Carrillo-Larco[1,2,3]

Manuel Castillo-Cara[4,5]

Jose Francisco Hernández Santa Cruz[6]

1. Department of Epidemiology and Biostatistics, School of Public Health, Imperial College London, London, UK
2. CRONICAS Centre of Excellence in Chronic Diseases, Universidad Peruana Cayetano Heredia, Lima, Peru
3. Universidad Continental, Lima, Peru
4. Ontology Engineering Group, Universidad Politécnica de Madrid, Madrid, Spain
5. Universidad de Lima, Lima, Peru
6. Independent researcher, Edinburgh, UK

Corresponding author:

Rodrigo M Carrillo-Larco, MD
Department of Epidemiology and Biostatistics
School of Public Health
Imperial College London
rcarrill@ic.ac.uk

For peer review only

For peer review only - http://bmjopen.bmj.com/site/about/guidelines.xhtml

ABSTRACT

Objectives: During the COVID-19 pandemic, convolutional neural networks (CNNs) have been used in clinical medicine (e.g., chest X-rays classification). Whether CNNs could inform the epidemiology of COVID-19 classifying street images according to COVID-19 risk is unknown, yet it could pinpoint high-risk places and relevant features of the built environment. In a feasibility study, we trained CNNs to classify the area surrounding bus stops (Lima, Peru) into *moderate* or *extreme* COVID-19 risk.

Design: CNN analysis based on images from bus stops and the surrounding area. We used transfer learning and updated the output layer of five CNNs: NASNetLarge, InceptionResNetV2, Xception, ResNet152V2, and ResNet101V2. We chose the best performing CNN which was further tuned. We used GradCam to understand the classification process.

Setting: Bus stops from Lima, Peru. We used five images per bus stop.

Primary and secondary outcome measures: Bus stop images were classified according to COVID-19 risk into two labels: *moderate* or *extreme*.

Results: NASNetLarge outperformed the other CNNs except in the recall metric for the *moderate* label and in the precision metric for the *extreme* label; the ResNet152V2 performed better in these two metrics (85% vs 76% and 63% vs 60%, respectively). The NASNetLarge was further tuned. The best recall (75%) and F1 score (65%) for the *extreme* label were reached with data augmentation techniques. Areas close to buildings or with people were often classified as extreme risk.

Conclusions: This feasibility study showed that CNNs have the potential to classify street images according to levels of COVID-19 risk. In addition to applications in clinical medicine, CNNs and street images could advance the epidemiology of COVID-19 at the population level.

Key words: machine learning; deep learning; artificial intelligence; computer vision; built environment; population health.

For peer review only

Strengths and limitations of this study

- We used five images per bus stop and the outcome information was provided by an official Government institution.
- We leveraged on five well-known convolutional neural networks (transfer learning).
- The analysis focused on street images from one city only.
- Original data (street images) cannot be shared because of restricted access.

For peer review only

For peer review only - http://bmjopen.bmj.com/site/about/guidelines.xhtml

INTRODUCTION

In COVID-19 research, deep learning tools applied to image analysis (i.e., computer vision) have informed the diagnosis and prognosis of patients through classification of chest X-ray and computer tomography images.[1-3]These tools have helped practitioners treating COVID-19 patients.

On the other hand, the application of computer vision to study the epidemiology of COVID-19 has been limited. One relevant example is the use of Google Street View images to extract features of the built environment and associate these with COVID-19 cases in the United States of America.[4] This work showed that unstructured and non-conventional data sources, such as street images, can deliver relevant information to characterize the epidemiology of COVID-19 at the population level.[4] In a similar vein, though not exclusively addressing COVID-19, other researchers have leveraged on street images to study health-related social inequalities,[5] air polution,[6] walkability,[7] as well as the built environment and health outcomes.[8,9] These examples show the potential of computer vision for population health research, above and beyond its multiple applications in clinical medicine with diagnostic and prognostic models.

However, to the best of our knowledge, computer vision models to classify street images based on their COVID-19 risk do not exist. From a public health perspective, such models could be relevant to understand unique local features of the built environment related to high COVID-19 risk. In addition, these models could be applied to places where observed data are not available to identify whether this place is at moderate or high risk of COVID-19 and inform potential interventions. This could be particularly helpful in Low- and Middle-income countries where limited resources do not allow massive COVID-19 testing, leaving places with no observed information about the COVID-19 epidemiology, though the local epidemiology could be estimated based on available images or alternative sources.

In this pilot feasibility study, we aimed to ascertain whether a convolutional neural network (deep learning) model could classify street images of bus stops according to their COVID-19 risk (binary outcome: *moderate* versus *extreme* risk) in Lima, Peru. We also aimed to understand what features of the images were most influential in the classification process.

For peer review only

For peer review only - http://bmjopen.bmj.com/site/about/guidelines.xhtml

METHODS

Study design

We used convolutional neural networks to study street images of bus stops and their surroundings in Lima, Peru. We implemented a classification model to classify the bus stops into two labels: *moderate* or *extreme* risk of COVID-19. We addressed a classification problem.

Rationale

We used five images per bus stop covering 360 degrees around the bus stop. Therefore, we targeted the bus stop and the surrounding area. We did not target the bust stop itself alone. The bus stop was the anchor for the outcome label (*moderate* or *extreme* risk of COVID-19) in the immediate surrounding area. It is unlikely that COVID-19 risk would be confined to the bus stop itself. Rather, the bus stop would be a proxy of the risk in the immediate nearby area.

We combined the five images before randomly splitting into the train, test, and validation dataset. We used the function *train_test_split* which randomly splits the data with equal distribution of the target outcome. We did not condition the random split on the bus stops because we did not target the bus stop itself only. A random split would provide data to have different profiles of the built environment, green areas, bus stops, and other street features relevant for the model to learn and classify according to COVID-19 risk.

We deemed this a pilot feasibility proof-of-concept study because we aimed to provide preliminary data on whether convolutional neural networks could classify street images according to COVID-19 risk. While there is evidence about convolutional neural networks being used for classification of X-rays and other clinical images for COVID-19 diagnosis,[1-3] there is less evidence on convolutional neural networks being used for population health and COVID-19. Future research could leverage on this idea with more images, classifying into multiple outcome labels, and implementing more sophisticated networks.

Public health and epidemiological research usually rely on structured data sources such as health surveys and measurements from patients including samples such as blood. Unstructured data sources, such as images, are gaining attention in clinical medicine and have been used to develop diagnosis and prognostic models; however, the use of images, including street images, in public health and epidemiological research

For peer review only - http://bmjopen.bmj.com/site/about/guidelines.xhtml

is limited. This work elaborates on this premise and on the current burden by COVID-19 and was conceived to study whether street images can ascertain the COVID-19 risk in the community. If successful, a deep learning model to classify street images according to COVID-19 risk could be used for disease surveillance, and to estimate the risk in places where observed data lack.

Data sources

The labels (observed data) of the bus stops were downloaded from the website of the Authority for Urban Transport in Lima and Callao (*Autoridad de Transporte Urbano para Lima y Callao* (ATU), name in Spanish). This government office manages the public transportation service in Lima, and publishes a classification map in which all bus stops in Lima are set into four categories of COVID-19 risk: moderate < high < very high < extreme.[10] Although this is an official source of information from a government branch, details of how the bus stops were classified are not available; please, refer to the discussion section where we further elaborate on this caveat. In this pilot feasibility study, we only worked with the bus stops deemed as *moderate* (label 0) and *extreme* (label 1) risk of COVID-19. We used the classification profile released on 2021-05-24.[11] We conducted a pilot feasibility study considering two outcome labels only. This, because we aimed to ascertain whether our hypothesis was possible and lead to relevant results while studying, from a public health perspective, the most important outcomes signalling the extremes of the risk distribution. Developing a model to identify areas at moderate risk could signal places where restrictions can be relaxed or suspended. Similarly, developing a model to identify areas at extreme risk would signal places where restrictions should be kept or strengthened. Therefore, a model focusing on two labels only, where these labels represent the extremes of the risk distribution, would be relevant and provide actional evidence. Our study could demonstrate that convolutional neural networks could successfully classify street images according to COVID-19 risk, with not addition information such as number of cases or health determinants. This has not been studied before. Future work will leverage on this preliminary experience to develop a four-outcome model, using larger datasets and incorporating more sophisticated networks.

We used the location (longitude and latitude coordinates) of the bus stops to download their street images through the application programming interface (API) of Google Street View. That is, we downloaded all the images in one batch through the API, rather than each one at the time through the API or from the standard

For peer review only - http://bmjopen.bmj.com/site/about/guidelines.xhtml

Google Street View website. For each bus stop (i.e., from each coordinate), we downloaded five images: when the camera was facing at 0 degrees, at 90 degrees, at 180 degrees and at 270 degrees; in addition, we also downloaded one image in which the direction of the camera was not specified (i.e., the heading parameter in the API request was set at default). In other words, for each bus stop we had five images. We did this to maximise the available data and to cover the surrounding area of the bus stop.[12] Our rationale was that the bus stop itself would not be responsible for the classification (moderate or extreme risk), but the whole nearby environment. Consequently, if the bus stop was labelled as moderate or extreme risk, the same label applied to the images of the surrounding area. For example, if bust stop X was labelled as moderate risk, all five images for such bus stop were labelled as moderate risk (i.e., image of the bus top itself plus the four images of the surrounding area).

Original dataset

Overall, after downloading both the labels and the images, there were 1,788 bus stops with their corresponding label: 1,173 in the *moderate* category and 615 in the *extreme* category (1,173 + 615 = 1,788). Because we used five images per bus stop, the analysis included 8,940 (1,788 x 5) images and their corresponding label. The training dataset included a random sample of 60% (5,364) of the original dataset. As further explained in the next section (Data preparation and class imbalance), after correcting for class imbalance by introducing duplicates of the class with fewer observations, the training data included 7,024 observations (3,519 for *moderate* and 3,505 for *extreme* labels). The validation and test datasets included a random sample of 20% of the original dataset each (0.20 x 8,940 = 1,788); the validation and test datasets were not corrected for class imbalance.

Data preparation and class imbalance

We combined the images and the labels in one dataset, which was further divided into three datasets: the training dataset including 60% of the data, the validation dataset including 20%, and the test dataset including the remaining 20%. Data allocation to each of these three datasets was at random. After splitting the data, we corrected for class imbalance in the train dataset only. We randomly multiplied the number of images in the imbalanced outcome by 0.9. This led to virtually the same number of images for the *moderate* and *extreme* risks labels.

For peer review only - http://bmjopen.bmj.com/site/about/guidelines.xhtml

There were two outcomes of interest: *moderate* and *extreme* risk. However, there were more observations in the *moderate* category than in the *extreme* category. That is, there was class imbalance. After splitting the data into the training, test, and validation sets, we corrected for class imbalance in the training dataset only. We randomly increased the number of observations in the extreme category by 90% in the training dataset (not in validation and test datasets). The original (before correction for class imbalance) training set had 3,519 observations in the *moderate* category and 1,845 in the *extreme* category (3,519 + 1,845 = 5,346). After correcting for class imbalance as described before, the training dataset had 3,519 observations in the *moderate* category (this number did not change) and 3,505 (1.9 x 1,845) observations in the *extreme* category. Therefore, there were 3,519 (*moderate*) + 3,505 (*extreme* after class imbalance correction) = 7,024 images and labels in total in the training dataset.

Analysis

In-depth details about the analysis are available in Supplementary Materials pp. 03-06. The analysis code (Python Jupyter notebooks) is also available as Supplementary Materials.

In brief, in a pre-specified protocol we decided to elaborate on five deep convolutional neural networks pre-trained with ImageNet (i.e., transfer learning). We chose these five networks because they have the best top-5 accuracy of all models available in the Keras library:[12] NASNetLarge, InceptionResNetV2, Xception, ResNet152V2 and ResNet101V2. We implemented these five models with the same hyper-parameters, and then we selected the one with the best performance which was further tuned and tested. The image classification model was based on the latter model only (i.e., the one with the best performance out of the five candidate models). We reported the loss and accuracy in the validation and test datasets; we also used the test dataset to report the accuracy, recall and F1 score for each of the two possible outcomes (*moderate* or *extreme* risk). Finally, we used GradCam (class activation maps) to identify which areas of the input image were more relevant to inform the classification process;[13] for this, we randomly selected four images per outcome (i.e., four images from the *moderate* label and four images from the *extreme* label). Areas most activated as shown by brighter colours, would be decisively in the classification process.

Patient and public involvement

Human subjects did not participate nor were involved in this study.

For peer review only - http://bmjopen.bmj.com/site/about/guidelines.xhtml

RESULTS

Selection of the pre-trained model out of five candidate models

We used transfer learning and updated the output layer of five convolutional neural networks to predict our two classes of interest. The NASNetLarge architecture and weights outperformed the other candidate convolutional neural networks, except in the recall metric for the *moderate* label: 76% versus 85% in NASNetLarge and ResNet152V2, respectively (Table 1). The ResNet152V2 also performed better than the NASNetLarge in the precision metric for the *extreme* label (60% vs 63%). Further experiments were only conducted with NASNetLarge because, overall, it performed better than the other pre-trained networks.

For peer review only

For peer review only - http://bmjopen.bmj.com/site/about/guidelines.xhtml

Table 1: Performance of the five candidate convolutional neural networks

	NASNetLarge	InceptionResNetV2	Xception	ResNet152V2	ResNet101V2
**Loss, validation**	0.526799	0.554040	0.533278	0.793147	0.744385
**Accuracy, validation**	0.742046	0.713636	0.730682	0.721023	0.723295
**Loss, test**	0.539906	0.557637	0.555917	0.800661	0.726274
**Accuracy, test**	0.731818	0.721591	0.706818	0.722727	0.718750
**Precision, label 0 (*moderate*)**	0.82	0.78	0.82	0.76	0.80
**Recall, label 0 (*moderate*)**	0.76	0.81	0.71	0.85	0.76
**F1 score, label 0 (*moderate*)**	0.79	0.79	0.76	0.80	0.78
**Precision, label 1 (*extreme*)**	0.60	0.61	0.56	0.63	0.58
**Recall, label 1 (*extreme*)**	0.68	0.56	0.70	0.48	0.64
**F1 score, label 1 (*extreme*)**	0.64	0.58	0.62	0.54	0.61

Green colour highlights the best metric, yellow colour highlights the second best metric and red colour highlights the third best metric row-wise. The precision, recall and F1 score are presented as proportions (multiply by 100 to have percentages). The precision, recall and F1 score were computed with the test dataset. Receiver Operating Characteristic (ROC) Curves for each model are available in Supplementary Materials.

For peer review only - http://bmjopen.bmj.com/site/about/guidelines.xhtml

202 **Model performance**

203 We further tuned NASNetLarge with different hyper-parameters aiming to improve the accuracy (Table 2).

For peer review only

For peer review only - http://bmjopen.bmj.com/site/about/guidelines.xhtml

Table 2: Further tuning of the selected model (NASNetLarge) and the performance metrics

		New model specifications			
	Original model (as in Table1)	horizontal_flip = True // epochs = 25 (stopped at 12)	horizontal_flip = True // zoom_range = 0.30 // epochs = 25 (stopped at 15)	decay = 0.1/10 // epochs = 25 (stopped at 12)	decay = 0.1/10 // factor = 0.3 // epochs = 25 (stopped at 12)
**Loss, validation**	0.526799	0.534797	0.537553	0.532246	0.532246
**Accuracy, validation**	0.742046	0.737500	0.739773	0.732386	0.732386
**Loss, test**	0.539906	0.550286	0.528204	0.538252	0.538252
**Accuracy, test**	0.731818	0.719318	0.735795	0.725568	0.725568
**Precision, label 0 (*moderate*)**	0.82	0.85	0.76	0.83	0.83
**Recall, label 0 (*moderate*)**	0.76	0.71	0.87	0.74	0.74
**F1 score, label 0 (*moderate*)**	0.79	0.77	0.81	0.78	0.78
**Precision, label 1 (*extreme*)**	0.60	0.57	0.66	0.59	0.59
**Recall, label 1 (*extreme*)**	0.68	0.75	0.47	0.71	0.71
**F1 score, label 1 (*extreme*)**	0.64	0.65	0.55	0.64	0.64

Green colour highlights the best metric, yellow colour highlights the second best metric and red colour highlights the third best metric row-wise considering only the new model specifications. The precision, recall and F1 score are presented as proportions (multiply by 100 to have percentages). Receiver Operating Characteristic (ROC) Curves for each model are available in Supplementary Materials.

First, building on the initial hyper-parameters, we implemented two data augmentation options: horizontal flip and zoom range. We chose these two data augmentation methods because they appropriately fit the images under analysis; for example, because we were working with street images, a vertical flip would not seem appropriate. The new model with horizontal flip improved the recall and F1 score for the *extreme* label; from 68% with the original NASNetLarge to 75%, and from 64% to 65% (Figure 1). The new model with horizontal flip and zoom range at 30% had better performance that the original NASNetLarge model in six out of ten parameters, including precision for the *extreme* label.

Second, also building on the initial hyper-parameters (i.e., without data augmentation), the decay in the stochastic gradient descendent optimizer was changed from 1/25 (25 was the number of epochs) to 1/10 (the number of epochs was not changed). This model did not substantially improve the performance of the model.

Third, building on the last specification (i.e., model with a decay of 1/10), we updated the monitoring factor which updated the learning rate when it did not improve through epochs. Originally, this factor was 0.1, and we updated it to 0.3. This model did not substantially improve the performance of the model.

GradCam

In the GradCam (i.e., class activation maps) analysis we used the NASNetLarge model with one data augmentation technique (horizontal flip). Even though the performance of the NASNetLarge model with two data augmentation techniques (horizontal flip and zoom range) was better in more metrics, the model with horizontal flip only had better recall and F1 score for the *extreme* label. The main indications for a *moderate* risk classification were the presence of green areas and lack of close nearby buildings. That is, images with several open spaces like parks, open streets, or wide avenues, would most likely be classified as moderate risk. Conversely, areas close to buildings, with a considerable presence of people, and with meeting points (e.g., street vendors), were often classified as extreme COVID-19 risk. In other words, bus stops with one or multiple street vendors, newspapers stand, or any other point for people to gather around, would most likely be classified as extreme risk. The presence of cars did not seem to impact the classification process.

For peer review only

For peer review only - http://bmjopen.bmj.com/site/about/guidelines.xhtml

DISCUSSION

Main findings

With almost all research on computer vision and COVID-19 focusing on diagnostic models based on X-rays and other clinical images, our work is novel because it borrows techniques from computer vision into epidemiology and population health leveraging on available data (street images). In this study we showed that deep convolutional neural networks can classify street images according to their COVID-19 risk with acceptable accuracy. Future work should strengthen available convolutional neural networks or develop a new architecture which could maximize the accuracy classification, not only for a binary outcome but also covering multiple outcomes. This work could spark interest to use convolutional neural networks –and other artificial intelligence tools– to advance population health and the epidemiological knowledge of COVID-19 (and other diseases), above and beyond the applications of convolutional neural networks for diagnosis and prognosis of individual patients (e.g., classification of chest X-rays and compute tomography images[1-3]).

Results in context

This work signalled that a deep neural network is moderately accurate to classify street images according to COVID-19 risk levels. These results are encouraging because the task we pursued was difficult: to classify street images into levels for which there is no unique intrinsic information in the images. Classification of, for example, chest X-ray images into healthy or ill could be easier for a convolutional neural network because the X-ray of someone with a disease (e.g., pneumonia) would have unique features (e.g., infiltrate spots at the bottom of the lungs) that a chest X-ray of someone healthy would not have at all. Conversely, in our case, the street images did not have a unique underlying pattern to guide the classification process. Our model had to work harder to find those unique characteristics to decide between *moderate* and *extreme* risk.

Further tuning of the selected model (NASNetLarge) suggested that data augmentation methods improved the performance of the model. When we updated the learning rate optimizer (decay and factor parameters), the model performance did not substantially improve. This could suggest that, for this particular task, we may need a large number of images. Alternatively, several combinations of data augmentation techniques

For peer review only

For peer review only - http://bmjopen.bmj.com/site/about/guidelines.xhtml

261 would need to be tested. Data augmentations should be carefully considered to select those most suitable
262 for these images; for example, vertical flip may not be a reasonable choice for street images.

263 Nguyen and colleagues used Google Street View images to associate features of the built environment with
264 COVID-19 cases in several states in the United States of America.[4] Although we could have followed the
265 same approach, there would be some unique local features of the built environment that may not have been
266 identified by available object detection tools (e.g., street vendors and newspaper stands). We are not aware
267 of other peer-reviewed papers in which street images have been classified according to COVID-19
268 outcomes developing a new model or leveraging on transfer knowledge from an established neural network.
269 Our work contributes to the available literature with a newly trained model benefiting from transfer learning
270 from a large and well-known architecture (NASNetLarge), based on images from a city in an upper-middle
271 income country (Lima, Peru).

272 The activation maps (GradCam results) are not only useful to analyse the model's interpretation capability,
273 but they bolster the existing evidence of crowded places or indoor venues (such as nearby buildings) as
274 COVID-19 high-risk areas. For example, areas with street vendors would activate more than open spaces
275 for the extreme risk classification; on the other hand, open areas would play a major role in classifying
276 moderate risk images. Overall, our findings agree with the evidence describing crowded areas, such as
277 restaurants, gyms, hotels, and cafes, as having high COVID-19 transmission risk.[14] Furthermore, our work
278 advances the field by showing that street images with no other clinical or epidemiological data, have
279 moderate accuracy to predict COVID-19 risk.

280 **Public health implications**

281 Our work could have pragmatic applications to better understand the epidemiology of COVID-19 and to
282 inform public health interventions. For example, our model –and future work improving this analysis– could
283 be used to characterize bus stops and other public places for which labelled data are not available. We
284 worked with images from bus stops in Lima, and our model could be applied to bus stops in other cities to
285 characterize their COVID-19 risk, particularly where observed data are not available. Furthermore, our work
286 could spark interest to conduct more sophisticated analyses, like semantic segmentation whereby some
287 unique elements of the local environment could be identified as potential high-risk places. For example, bus

For peer review only - http://bmjopen.bmj.com/site/about/guidelines.xhtml

stops in Lima often host food street vendors and newspaper stands where people usually gather. Perhaps, the bus stops themselves are not high-risk places, but those surrounding shops. This could inform policies and interventions to reduce the COVID-19 risk in these places. Overall, deep learning techniques, including convolutional neural networks, could be adopted by epidemiological research to advance the evidence about risk factors as well as disease outcomes and distribution, in addition to their current use in clinical medicine.[1-3]

Our work was designed to understand whether and how well street images, without complementary data, can predict COVID-19 risk. Our results support the idea that the built environment alone is a health determinant because the street images were not complemented with other epidemiological data such as number of cases or COVID-19 transmission. Measuring COVID-19 throughout a country can be challenging and barriers include lack of access to tests as well as laboratory facilities to process the samples, and limited health or trained personnel to take the samples. Our work suggests that street images could serve as proxy to estimate the COVID-19 risk in places where this information does not exist based on observed data. Therefore, we provide preliminary evidence suggesting that street images can be instrumental in COVID-19 surveillance.

Finally, as argued before, this is a pilot feasibility proof-of-concept study to study whether convolutional neural networks could classify street images according to COVID-19 indicators. This work complements the current use of convolutional neural networks for COVID-19 classification of clinical images (e.g., X-rays). This work should be regarded as the first step in the use of convolutional neural networks in epidemiology and population health relevant to COVID-19; this work is not the ultimate work on this subject and future research should improve our approach and results.

Ongoing and future work

Ongoing and future work includes the development of a classification model for the four outcome labels (i.e., *moderate*, *high*, *very high* and *extreme* COVID-19 risk). We will implement techniques that can potentiate the classification capacity of the neural networks, including ensemble models,[15] novel loss functions not currently implemented in the Keras environment (e.g., squared earth mover's distance-based loss

For peer review only - http://bmjopen.bmj.com/site/about/guidelines.xhtml

function),[16] and we may try other architectures (e.g., SqueezeNet[17]) with similar precision yet less computationally expensive. Because most of our bus stop images also depicted buildings, we may try to use a network already trained on images of buildings and other city landscapes (e.g., Places-365).

Strengths and limitations

We followed a pre-defined protocol which included transfer learning leveraging on large and deep neural networks trained with millions of images (ImageNet). We still had to train the parameters of the output layer, for which we did not have a massive number of images. Future work could expand our analysis with information and images from more bus stops or other public spaces to train a more robust model. Ideally, these images should come from different cities. This information may be available in other countries. There are further limitations we must acknowledge. First, the images and labels were not synchronic; that is, the figures and the labels were not collected on the same date. This is a shared limitation with other studies working with street images from open sources (e.g., Google Street View), because these images are not taken continuously or in real time. This should not be a major limitation because the analysis mostly focused on the built environment which has not changed substantially in recent years. Because this feasibility study showed that the classification model performed moderately well, researchers could collect new images in a prospective work to verify our findings with synchronic data. In this line, satellite images collected daily could be useful. Second, we did not have exact details on how the bus stops were classified by the local authorities. Nevertheless, we used official information which is provided to the public for their safety and to inform them about the progression of the COVID-19 pandemic. Because it is an official source of public information, we trust their method for classification is sound and based on the best available evidence. This limitation should not substantially bias our model or results because the labels were clearly available from the data provider (transport authority), and we did not have to make any assumptions nor manual labelling. However, this may limit the external reproducibility of our work because other researchers may not label their images following the same criteria by our data source. We argue that this should not rest importance to our work because which could serve as basis for future research in the area in which the underlying labelling criteria are clearer. Third, we had five images per bus stop: the fifth image did not look at a specific angle, unlike the other four images that looked at 0, 90, 180 and 270 degrees around the bus stop. Therefore, the fifth image had some overlap with the other images. We took this decision to maximize the

For peer review only - http://bmjopen.bmj.com/site/about/guidelines.xhtml

available data. Researchers with access to more labelled information, perhaps from public places overseas, could use the four images without overlap and not significantly reducing the dataset size. In this line, the datasets (training, test, and validation) were split randomly and, just by chance albeit improbably, all images of one particular bus stop could have fallen in a subset (e.g., test dataset). If so, the model would have poor accuracy to predict this specific bus stop because the model did not have any information/images about that bus stop in the training dataset. However, because we trained the model to classify *moderate* and *extreme* risk of COVID-19, the model learnt patterns and profiles of the bus stops and their surrounding areas. This training could then be applied to other bus stops with similar characteristics. The GradCam analysis helped us to exemplify the patterns most influential in the selection process. Arguably, the influential patterns would be in all or most images. Fourth, our model cannot be independently reproduced because we could not make the underlying data available because these images do not belong to us. Google Street View images are available through the API, though they need personal login credentials. Although this would not replace the raw underlying data, to increase the transparency of our work we made available the Jupyter notebooks used in the analysis (Supplementary Materials). These notebooks show the codes and results. Fifth, we did not report or discuss the algorithms or computations behind the convolutional neural networks we used for transfer learning. As per our protocol, we chose and applied a set of established convolutional neural networks to solve a classification problem. Disentangling the underlying mechanisms underneath each convolutional neural network was beyond the scope of this work. Nevertheless, it is relevant to understand the areas of the images most influential in the classification process. This way we can verify if the classification process followed a logical path. We therefore reported the GradCam analysis.

Conclusions

This study showed that a convolutional neural network has moderate accuracy to classify street images into *moderate* and *extreme* risk of COVID-19. In addition to applications in clinical medicine, deep convolutional neural networks have the potential to also advance the epidemiology of COVID-19 at the population level exploding unstructured and non-conventional data sources.

For peer review only - http://bmjopen.bmj.com/site/about/guidelines.xhtml

FIGURES

Figure 1: Confusion matrix for the best NASNetLarge model

This NASNetLarge model corresponds to the one with data augmentation of horizontal flip (first column in the new model specification section of Table 2). The figure shows the absolute number of images in each label: observed (true) on the y-axis and predicted on the x-axis.

For peer review only

For peer review only - http://bmjopen.bmj.com/site/about/guidelines.xhtml

CONTRIBUTIONS

RMC-L and JFHSC conceived the idea. RMC-L conducted the analysis with support from JFHSC and MC-C. MC-C supported the revised analysis. All authors approved the submitted version.

FUNDING

RMC-L is supported by a Wellcome Trust International Training Fellowship (Wellcome Trust 214185/Z/18/Z).

COMPETING INTERESTS

No conflict of interest.

DATA AVAILABILITY STATEMENT

Outcome (i.e., labels: moderate and extreme COVID-19 risk) data are available online: https://sistemas.atu.gob.pe/paraderosCOVID; this information was systematized at https://github.com/jmcastagnetto/lima-atu-covid19-paraderos. The images were downloaded from Google Street View through the API with a personal account; images cannot be shared with third parties. All analysis codes are available as Python Jupyter Notebooks in Supplementary Materials. JupyterLab Notebooks and the final model (weights) are available at:

https://figshare.com/articles/online_resource/Street_images_classification_according_to_COVID-19_risk_in_Lima_Peru_A_convolutional_neural_networks_feasibility_analysis/17321021

For peer review only

Ethical statement

Human subjects were not directly studied in this work. The analysed data are in the public domain and can be downloaded. The analysed data do not contain any personal identifiers. We did not seek approval by an ethics committee.

For peer review only

For peer review only - http://bmjopen.bmj.com/site/about/guidelines.xhtml

REFERENCES

1. Ghaderzadeh M, Asadi F. Deep Learning in the Detection and Diagnosis of COVID-19 Using Radiology Modalities: A Systematic Review. *Journal of healthcare engineering* 2021; **2021**: 6677314.

2. Mohammad-Rahimi H, Nadimi M, Ghalyanchi-Langeroudi A, Taheri M, Ghafouri-Fard S. Application of Machine Learning in Diagnosis of COVID-19 Through X-Ray and CT Images: A Scoping Review. *Frontiers in cardiovascular medicine* 2021; **8**: 638011.

3. Montazeri M, ZahediNasab R, Farahani A, Mohseni H, Ghasemian F. Machine Learning Models for Image-Based Diagnosis and Prognosis of COVID-19: Systematic Review. 2021; **9**(4): e25181.

4. Nguyen QC, Huang Y, Kumar A, et al. Using 164 Million Google Street View Images to Derive Built Environment Predictors of COVID-19 Cases. 2020; **17**(17).

5. Suel E, Polak JW, Bennett JE, Ezzati M. Measuring social, environmental and health inequalities using deep learning and street imagery. *Scientific reports* 2019; **9**(1): 6229.

6. Suel E, Sorek-Hamer M, Moise I, et al. What You See Is What You Breathe? Estimating Air Pollution Spatial Variation Using Street-Level Imagery. *Remote Sensing* 2022; **14**(14): 3429.

7. Nagata S, Nakaya T, Hanibuchi T, Amagasa S, Kikuchi H, Inoue S. Objective scoring of streetscape walkability related to leisure walking: Statistical modeling approach with semantic segmentation of Google Street View images. *Health & place* 2020; **66**: 102428.

8. Nguyen QC, Keralis JM, Dwivedi P, et al. Leveraging 31 Million Google Street View Images to Characterize Built Environments and Examine County Health Outcomes. *Public Health Rep* 2021; **136**(2): 201-11.

9. Nguyen QC, Sajjadi M, McCullough M, et al. Neighbourhood looking glass: 360º automated characterisation of the built environment for neighbourhood effects research. *Journal of epidemiology and community health* 2018; **72**(3): 260-6.

10. Autoridad de Transporte Urbano para Lima y Callao (ATU). Paraderos con Riesgo de COVID - 19. July 22, 2022. https://sistemas.atu.gob.pe/paraderosCOVID.

[dataset]11. jmcastagnetto. Data from: lima-atu-covid19-paraderos. July 22, 2022. https://github.com/jmcastagnetto/lima-atu-covid19-paraderos.

12. Keras applications. July 26, 2022. https://keras.io/api/applications/.

For peer review only - http://bmjopen.bmj.com/site/about/guidelines.xhtml

13. Selvaraju RR, Cogswell M, Das A, Vedantam R, Parikh D, Batra D. Grad-CAM: Visual Explanations from Deep Networks via Gradient-Based Localization. *International Journal of Computer Vision* 2020; **128**(2): 336-59.

14. Chang S, Pierson E, Koh PW, et al. Mobility network models of COVID-19 explain inequities and inform reopening. *Nature* 2021; **589**(7840): 82-7.

15. Ganaie M, Hu M. Ensemble deep learning: A review. *arXiv preprint arXiv:210402395* 2021.

16. Hou L, Yu C-P, Samaras D. Squared earth mover's distance-based loss for training deep neural networks. *arXiv preprint arXiv:161105916* 2016.

17. Iandola FN, Han S, Moskewicz MW, Ashraf K, Dally WJ, Keutzer K. SqueezeNet: AlexNet-level accuracy with 50x fewer parameters and< 0.5 MB model size. *arXiv preprint arXiv:160207360* 2016.

For peer review only

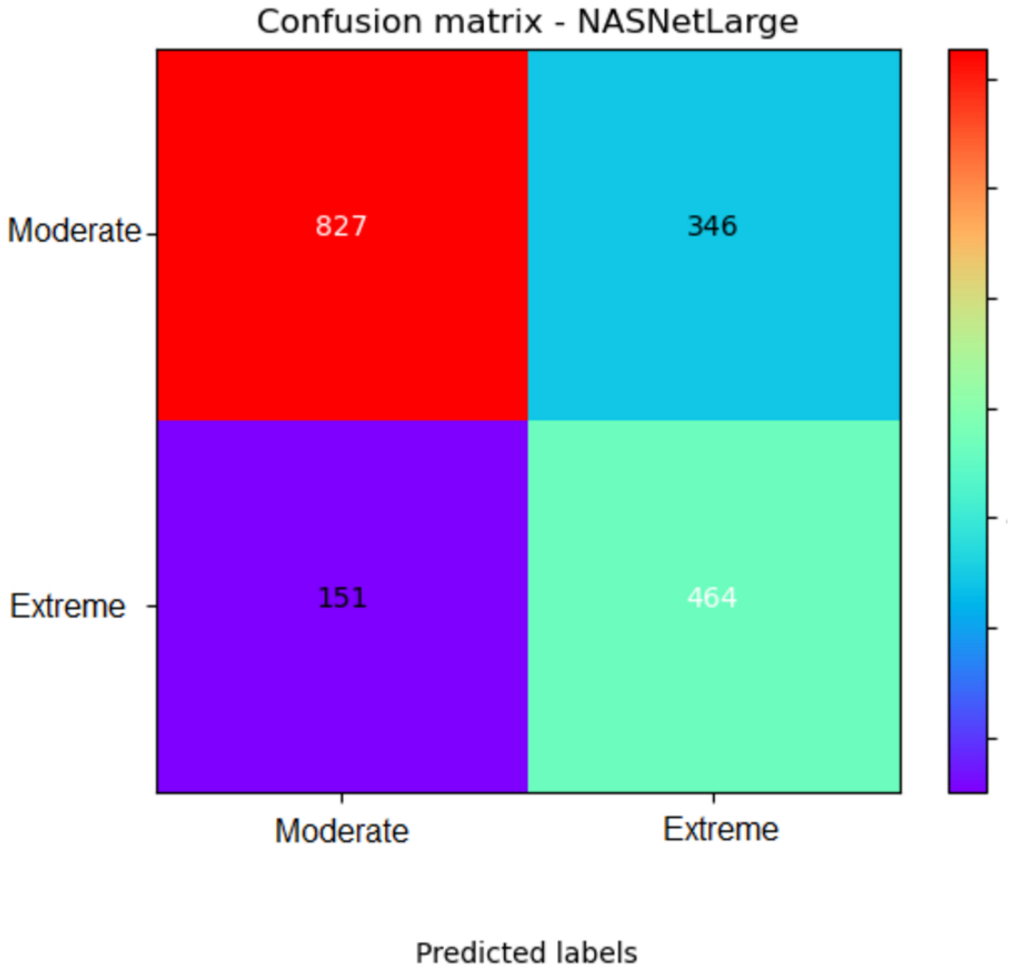

Figure 1: Confusion matrix for the best NASNetLarge model

This NASNetLarge model corresponds to the one with data augmentation of horizontal flip (first column in the new model specification section of Table 2). The figure shows the absolute number of images in each label: observed (true) on the y-axis and predicted on the x-axis.

601x601mm (38 x 38 DPI)

Supplementary Materials

Street images classification according to COVID-19 risk in Lima, Peru: A convolutional neural networks feasibility analysis

Corresponding author:

Rodrigo M Carrillo-Larco, MD

Department of Epidemiology and Biostatistics

School of Public Health

Imperial College London

rcarrill@ic.ac.uk

For peer review only

Contents

EXPANDED METHODS 3

Overview 3

System details 3

Images 3

Labels (outcome) 4

Class imbalance 4

Image data generator 4

Convolutional neural networks 5

Model Architecture 5

Model Training 6

Activation Heatmap by GradCam Visualization 7

References 7

Receiver Operating Characteristic (ROC) Curves I 9

Receiver Operating Characteristic (ROC) Curves II 10

For peer review only

For peer review only - http://bmjopen.bmj.com/site/about/guidelines.xhtml

EXPANDED METHODS

Overview

In a pre-specified protocol we decided to test five well-known convolutional neural network architectures. These five networks are those with the best accuracy among the models available in the Keras library.[1] From these five candidate models, we chose the one with the best performance for our task; this model was further tuned to improve its performance.

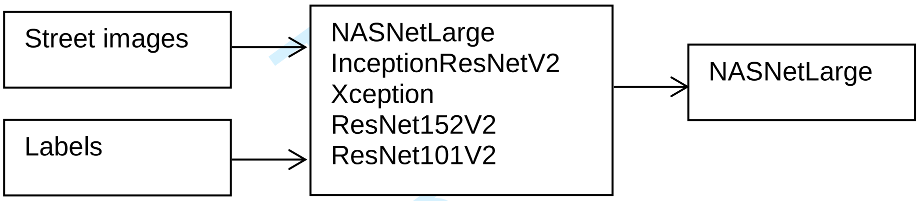

Analysis code (Jupyter notebooks) and the weights of the final model are found here: https://drive.google.com/file/d/1HXLsenn7yvxri7n2xE80WMtxQzj5fgBX/view?usp=sharing

System details

Analyses were conducted with a GPU NVIDIA Quadro P1000 on Python Jupyter (version 3.7.10). The notebooks are provided as supplementary materials.

Images

The list of bus stop in Lima, Peru, and their COVID-19 risk, are provided by local transport authority.[2] This information has been extracted and homogenized and is available online.[3] Key for our work, this information contains: i) location of each bus stop (latitude and longitude coordinates), which was used to extract street images; and ii) the COVID-19 risk level assigned to each bus stop, which was used as the outcome labels.

We downloaded the images from Google Street View through the application programming interface (API). We used the Python libraries *google_streetview.api* and *request*. For each bus stop (i.e., for each latitude and longitude coordinate), the API request specified the heading parameter = [0, 90, 180, 270]; in addition, we downloaded one image for which the heading parameter was set at default. Consequently, for each bus stop we had five images in total. The images were downloaded with size 640x640 pixels. In this feasibility study there were 1,788 bus stops, and because we had five images per bus stop, we used 8,940 (1,788 x 5) images in total.

For peer review only - http://bmjopen.bmj.com/site/about/guidelines.xhtml

Labels (outcome)

We used the bus stop classification released on 2021-05-24,[2, 3] by the local transport authority in Lima, Peru.[2] They classify the bus stops in four categories of COVID-19 risk: moderate, high, very high and extreme risk.[2] Details on how they made this classification are not available. Nevertheless, because this is official information released by a public authority to inform the general population, we trust their classification is based on the best available evidence. In this feasibility work we only used bus stops labelled as *moderate* (n=1,173) and *extreme* (n=615) COVID-19 risk. There were 1,788 (1,173 + 615) bus stops in total. The list of labels was appended five times, so that we would have as many images as labels (NB: we had five images per bus stop as described above).

Class imbalance

There were two outcomes of interest: moderate and extreme risk. However, there were more observations in the moderate category than in the extreme category. That is, there was class imbalance. After splitting the data into the training, test and validation sets, we corrected for class imbalance. We randomly increased the number of observations in the extreme category by 90% in the training dataset (not in validation and test datasets). The original (before correction for class imbalance) training set had 3,519 observations in the moderate category and 1,845 in the extreme category (3,519 + 1,845 = 5,346). After correcting for class imbalance as described before, the training dataset had 3,519 observations in the moderate category (this number did not change) and 3,505 (~1.9 x 1,845) observations in the extreme category (3,519 + 3,505 = 7,024 total sample in the training dataset).

Image data generator

We constructed a dataframe with two columns: the path to each image (i.e., to the exact location were the images were saved) and the corresponding label for each image. This dataframe was passed to the *ImageDataGenerator* function of the *keras.preprocessing.function* library. At this point, we also re-scaled the images between 0 and 1 by dividing by 255. Then, we created three image iterators: one for the training, validation and test datasets (*train_datagen.flow_from_dataframe* function). To the *train_datagen.flow_from_dataframe* function we passed the datarame with the location of the images and their labels, specified this was a categorical classification problem, a batch size of 32, and a

For peer review only - http://bmjopen.bmj.com/site/about/guidelines.xhtml

specific image size for each candidate model (see table below); in addition, the image iterator for the training dataset had the shuffle parameter as *True*.

Convolutional neural networks

We decided to train five candidate convolutional neural networks with the following specifications.

	NASNetLarge	InceptionResNetV2	Xception	ResNet152V2	ResNet101V2
**Image size**	331 x 331	299 x 299		224 x 224	
**Pre-trained weights**	ImageNet				
**Top layer included?**	No				
**Trainable parameters**	None				
**Additional layers**	Dense layer with 2 neurons (for the two outcome labels), with *softmax* activation				
**Number of epochs for training**	25				
**Optimizer**	SGD(learning_rate = 0.1, momentum = 0.9, decay = 0.1/number of epochs, nesterov = True)				
**Loss function**	Binary crossentropy				

SDG: stochastic descent gradient.

In addition, we monitored the validation loss: when the validation loss would not improve in one epoch, then the learning rate was multiplied by 0.1. We also specified an early stop: when the validation loss would not improve for ten epochs, the training would stop. To choose between the five candidate models we did not implement any data augmentation methods.

We chose the model with the best performance (Table 1 in the main text), which was further tuned (Table 2 in main text).

Model Architecture

The chosen model (NASNetLarge) presented in this article is a state-of-the-art convolutional neural network (CNN) pre-trained with the ImageNet dataset of images. The NASNetLarge neural network is widely used in computer vision. It is composed of several layers of convolutional cells that extract the most important features from each image, learning which features are the most characteristic from each image category. Most pre-trained state-of-the-art CNNs, such as the AlexNet or the ResNet50, base their innovations in the use of complex activation functions, efficient designs and special layers, such as the Batch Normalization,[4] which not only improve accuracy performance, but also reduce the time needed to train a model. The ResNet50 neural network, for example, introduces the concept of residual neural networks, layers that skip their connections to other layers by adding connection shortcuts, thus reducing backpropagation training time and better generalizing models.

For peer review only - http://bmjopen.bmj.com/site/about/guidelines.xhtml

However, most of these CNNs had complex design that was built by using trial-and-error methods, oblivious to modern state-of-the-art optimization methods such as the use of evolutionary and nature-inspired algorithms or Reinforcement Learning (RL). This is where Neural Search Architecture (NAS) Networks come in play.[5] Dubbed as a breakthrough in machine learning automation, NAS networks use *AI for AI.* The network's whole architecture is designed by optimization algorithms, such as Gradient-based search and RL. The idea behind this is that, setting the right restrictions to avoid repetitive inclusion of layers, the network cannot only be trained by its parameters, but by its own architecture, adding and changing layers, activations functions and connections.

Our research uses the NASNetLarge model available by Keras,[1] pre-trained on the ImageNet dataset on 1000 categories. Because we only have two categories to train on (moderate and extreme), we started by replacing the network's fully connected classification layer by a 2-neurons layer, with a softmax activation function.[1] The rest of the original NASNetLarge network was kept unmodified to preserve its proven architecture.

Model Training

To train our model we used transfer learning. Based on the pre-trained NASNet model, we retained its parameters and froze them, keeping just trainable the last fully connected layer, initializing its parameters randomly by He uniform initialization.[6] This single-stage training took advantage of the pre-trained parameters speeding up training by just focusing on the last decision layer. The model was trained for 30 epochs. Each epoch is understood as a complete training cycle through the whole train dataset. Data is then fed by batches; once all batches are loaded, an epoch is finished. By using a batch size of 32, training and testing sets are fed to the neural network. The loss function, as we are dealing with a binary classification model, is binary cross entropy.

As optimizer we used the Stochastic Gradient Descent (SGD).[7] One of the key advantages of this optimizer is due to its stochasticity in selecting each batch for the backpropagation step. Although the model takes longer to converge, unlike other optimizers, the SGD has been proven to converge better local optima, searching for global optima with much more easiness. This factor helps also to reduce overfitting.

For peer review only - http://bmjopen.bmj.com/site/about/guidelines.xhtml

Activation Heatmap by GradCam Visualization

Model interpretability is a feature that has gained importance since the appearance of methods such as GradCam.[8] This technique uses the values from the gradients in the model's final feature layer to produce visual explanations, highlighting regions of importance taken by the model to infer a given input. In other words, this technique informs which areas of the input figure were more relevant to make the final classification.

Regions that represent higher gradient values, accounting for most of the last layer activations and network's decision, are represented by being coloured closer to the red portion of the spectrum. By comparison, regions with the lowest activations, not adding much information to the network's final decision, appear as areas closer to the blue portion of the spectrum. These gradient values are taken from the network's last convolutional layer, right before the last pooling layer.

References

1. Keras applications. URL: https://keras.io/api/applications/.
2. Autoridad de Transporte Urbano para Lima y Callao (ATU). Paraderos con Riesgo de COVID - 19. URL: https://sistemas.atu.gob.pe/paraderosCOVID.
3. URL: https://github.com/jmcastagnetto/lima-atu-covid19-paraderos.
4. Ioffe S, Szegedy C. Batch Normalization: Accelerating Deep Network Training by Reducing Internal Covariate Shift. *arXiv e-prints* 2015: arXiv:1502.03167.
5. Kyriakides G, Margaritis K. An Introduction to Neural Architecture Search for Convolutional Networks. *arXiv e-prints* 2020: arXiv:2005.11074.
6. He K, Zhang X, Ren S, Sun J. Delving Deep into Rectifiers: Surpassing Human-Level Performance on ImageNet Classification. *arXiv e-prints* 2015: arXiv:1502.01852.
7. Loshchilov I, Hutter F. SGDR: Stochastic Gradient Descent with Warm Restarts. *arXiv e-prints* 2016: arXiv:1608.03983.
8. Selvaraju RR, Cogswell M, Das A, Vedantam R, Parikh D, Batra D. Grad-CAM: Visual Explanations from Deep Networks via Gradient-Based Localization. *International Journal of Computer Vision* 2020; **128**(2): 336-59.

For peer review only

For peer review only

For peer review only - http://bmjopen.bmj.com/site/about/guidelines.xhtml

Receiver Operating Characteristic (ROC) Curves I

InceptionResNetV2

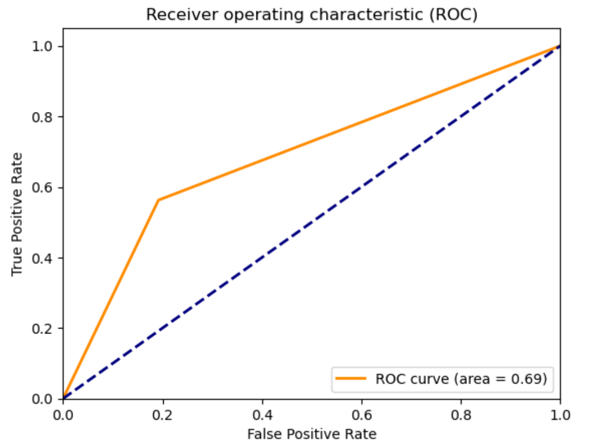

Xception

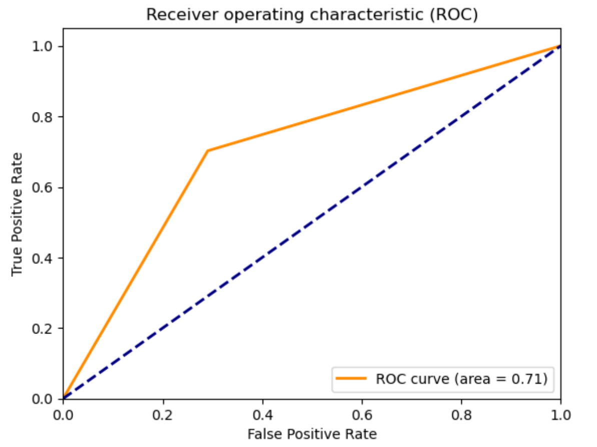

ResNet152V2

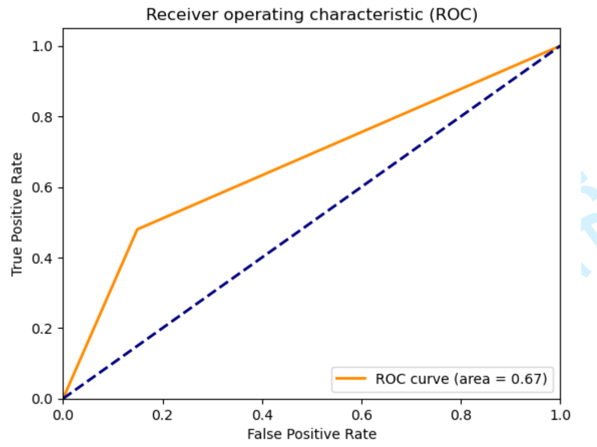

ResNet201V2

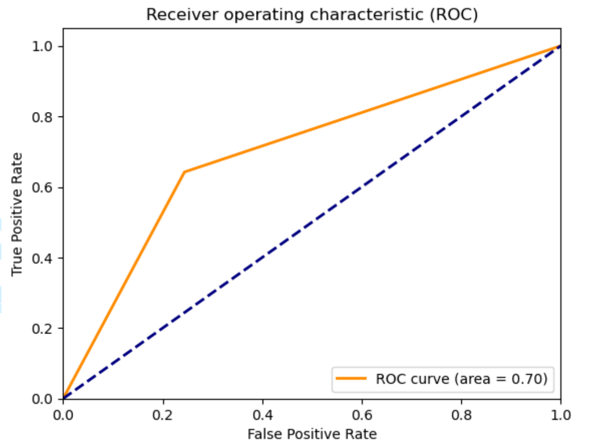

NASNetLarge

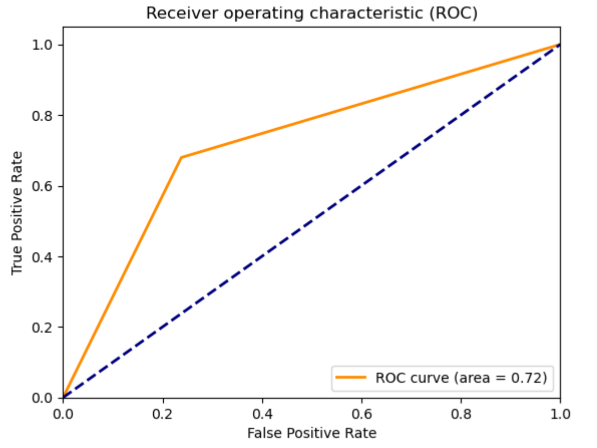

For peer review only - http://bmjopen.bmj.com/site/about/guidelines.xhtml

Receiver Operating Characteristic (ROC) Curves II

NASNetLarge [horizontal_flip = True // epochs = 25]

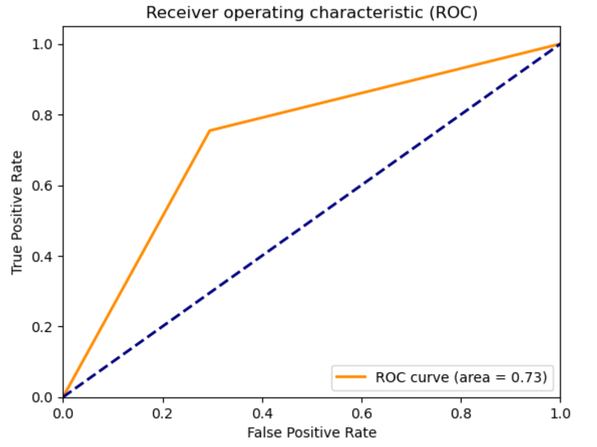

NASNetLarge [horizontal_flip = True // zoom_range = 0.30 // epochs = 25]

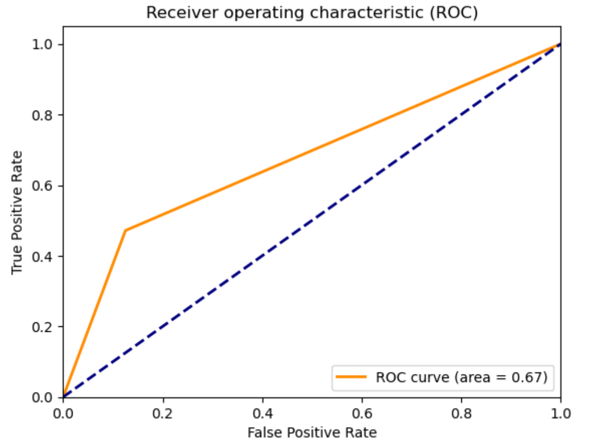

NASNetLarge [decay = 0.1/10 // epochs = 25]

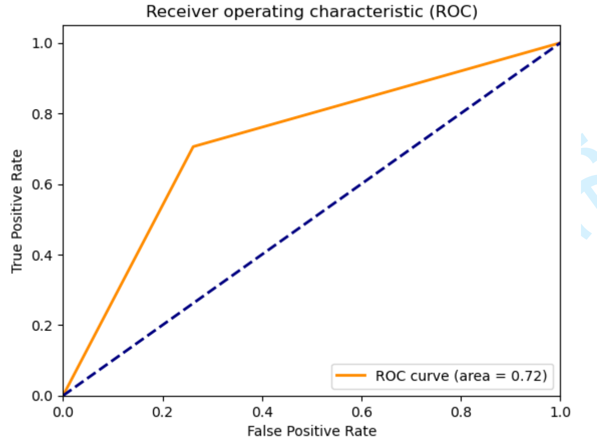

NASNetLarge [decay = 0.1/10 // factor = 0.3 // epochs = 25]

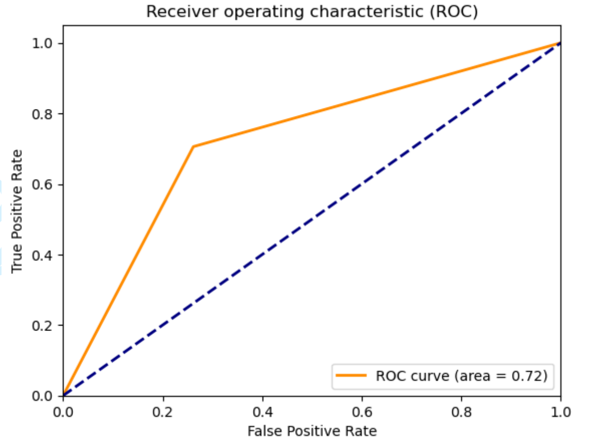

For peer review only - http://bmjopen.bmj.com/site/about/guidelines.xhtml
